# Engineered Cancer Nanovaccines: A New Frontier in Cancer Therapy

**DOI:** 10.1007/s40820-024-01533-y

**Published:** 2024-09-30

**Authors:** Yijie Wang, Congrui Liu, Chao Fang, Qiuxia Peng, Wen Qin, Xuebing Yan, Kun Zhang

**Affiliations:** 1 Central Laboratory and Department of Medical Ultrasound, Sichuan Academy of Medical Sciences, Sichuan Provincial People’s Hospital, School of Medicine, University of Electronic Science and Technology of China, No. 32, West Second Section, First Ring Road, Chengdu, 610072 People’s Republic of China; 2 Department of Stomatology and Central Laboratory, School of Medicine, Shanghai Tenth People’s Hospital, Tongji University, NO. 301 Yan-Chang-Zhong Road, Shanghai, 200072 People’s Republic of China; 3 Jiangsu Provincial Innovation and Practice Base for Postdoctors, Suining People’s Hospital, Affiliated Hospital of Xuzhou Medical University, No.2, Bayi West Road, Suining, Xu Zhou, 221000 Jiangsu Province People’s Republic of China

**Keywords:** Cancer nanovaccines, Immunotherapy, Nanobiotechnology, Immune targets, Signaling pathway

## Abstract

Vaccinations are essential for preventing and treating disease, especially cancer nanovaccines, which have gained considerable interest recently for their strong anti-tumor immune capabilities. Vaccines can prompt the immune system to generate antibodies and activate various immune cells, leading to a response against tumor tissues and reducing the negative effects and recurrence risks of traditional chemotherapy and surgery. To enhance the flexibility and targeting of vaccines, nanovaccines utilize nanotechnology to encapsulate or carry antigens at the nanoscale level, enabling more controlled and precise drug delivery to enhance immune responses. Cancer nanovaccines function by encapsulating tumor-specific antigens or tumor-associated antigens within nanomaterials. The small size of these nanomaterials allows for precise targeting of T cells, dendritic cells, or cancer cells, thereby eliciting a more potent anti-tumor response. In this paper, we focus on the classification of carriers for cancer nanovaccines, the roles of different target cells, and clinically tested cancer nanovaccines, discussing strategies for effectively inducing cytotoxic T lymphocytes responses and optimizing antigen presentation, while also looking ahead to the translational challenges of moving from animal experiments to clinical trials.

## Highlights

- We classified the carriers that built cancer nanovaccines, discussed their diversified applications and coincidently compared their advantages and disadvantages.
- Various cellular targets that guide the design and engineering of cancer nanovaccines are categorized and their characteristics and benefits are highlighted.
- The clinical cases and encountered challenges in cancer nanovaccines are discussed, during which reasonable solutions and future research direction are provided.

## Graphical abstract

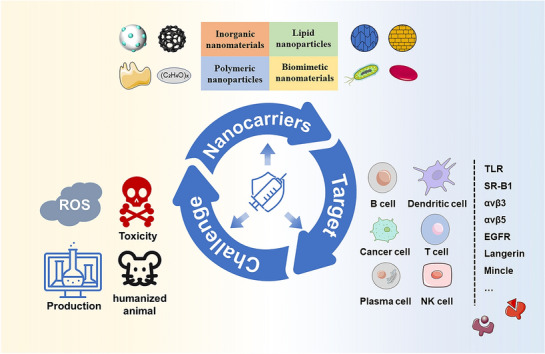

## Introduction

Vaccines, considered one of the great inventions in human medicine, induce robust immune responses by injecting antigenic substances [1], effectively preventing many life-threatening diseases such as smallpox, measles, and pertussis [1, 2]. Even the novel coronavirus of 2019 has witnessed the development of vaccines [3]. Some highly differentiated malignant tumors that are currently difficult to cure and prone to recurrence and metastasis, instill increasing fear and burden due to the high treatment costs and poor efficacy, driving the demand for developing novel cancer treatments. Consequently, scientists have shifted their focus to cancer immunotherapy that is often referred to as the fourth major modality for cancer treatment [4]. As a constituent of cancer immunotherapy, cancer nanovaccines utilize nanocarriers to deliver vaccine payloads into the body, exhibiting excellent anti-tumor immune effects and greatly accelerating progress in tumor prevention and treatment [5].

In this review, we systematically summarize various nanomaterial carriers used for vaccine fabrication, such as inorganic materials [6], lipid materials [7, 8], polymer materials [9, 10], viruses [11], and cell membranes from different cells [12, 13]. These nanomaterials possess favorable biocompatibility, adjuvant activity, and immunogenicity, but the preparation and storage of some materials can be challenging, and certain materials may exhibit inherent biotoxicity. Therefore, choosing the right nanocarriers is an essential factor in the development of vaccine formulations [14]. Additionally, we discuss and summarize the latest validated feasible cellular targets, including activating dendritic cells (DCs) to induce cellular immunity [15, 16], directly utilizing biomimetic DCs for T cell self-presentation [17, 18], hybrid immune strategies activating both T and B cells [19–22], and utilizing cancer cell membrane receptors for anticancer drug delivery. Subsequently, we pursue the clinical translation of these experiments and explore the current limitations and future prospects of development.

## What are *Cancer* Nanovaccines?

Vaccines are medications created to trigger immune reactions in the human body by introducing certain pathogens, like viruses or bacteria, to prevent the development of associated illnesses [23]. Typically, vaccines consist of one or more attenuated or inactivated forms including pathogenic microorganisms, or their toxins or surface proteins. After vaccination, the immune system identifies and memorizes these pathogens, and then elicits rapid and effective responses as the body encounters actual pathogens in the future, thereby preventing disease occurrence or mitigating its severity [1]. Various vaccines can be classified according to the specific pathogenic material they contain. Inactivated vaccines use pathogens that are no longer harmful, like viruses or bacteria, to stimulate the immune system and generate antibodies. Typical examples include influenza vaccines [24] and inactivated poliovirus vaccines (IPV) [25]. Attenuated vaccines employ weakened forms of pathogens, insufficient to cause disease but still capable of triggering immune responses. Typical examples included measles [26], mumps [27], and rubella vaccines [28]. Recombinant protein vaccines use specific surface proteins of pathogens rather than the entire pathogens, e.g., hepatitis B vaccines [29], and HPV vaccines [30]. Viral vector vaccines use other viruses as carriers, into which genes of the target pathogen are inserted such as adenovirus vectors in COVID-19 vaccines [31]. Nucleic acid vaccines activate immune responses by encoding the proteins of pathogens using mRNA or DNA. mRNA vaccines in COVID-19 vaccines belong to this category [31].

The high specificity and immunological memory exhibited by vaccines make them effective tools for treating diseases, prompting scientists to explore their use in cancer therapy. Novel cancer treatments in the form of cancer vaccines have been developed based on the previously mentioned types of vaccines. The goal of these vaccines is to trigger either humoral or cellular immune reactions by introducing antigens associated with tumors, ultimately prompting an immune reaction against cancer cells to help identify and eliminate them [32]. In 1980, the first cancer vaccines were created using tumor cells and tumor lysates to treat colorectal cancer [33]. Following this, the initial human tumor marker, MAGE-1 [34] was identified, leading to the introduction of cancer vaccines utilizing DCs in medical practice [35]. With the development of more cancer vaccines, personalized treatment and immunotherapy have been provided with new possibilities. However, as clinical trials progress, the limitations of cancer vaccines, such as low flexibility, insufficient immunogenicity, and poor specificity, have become increasingly apparent [23]. Biotechnologically produced cancer vaccines alone are no longer able to meet practical needs. As a new generation of products, cancer nanovaccines prepared using nanotechnology have demonstrated remarkable potential in tumor therapy. Compared to conventional vaccines, nanovaccines derived from tumors can provide a sustained source for all potential antigens, avoid antigen loss and effectively accomplish the screening of new antigens [36]. In addition, compared to conventional vaccines, nanovaccines, due to their special material coupling, can administer the antigens to more appropriate locations. The size and surface modifications of nanoparticles enable them to concentrate more readily in immune organs such as lymph nodes and spleen, thereby efficiently delivering antigens and adjuvants to target cells, enhancing the vaccine’s specificity and ensuring a stronger immune response [14]. Cancer nanovaccines can be designed to respond to specific physiological conditions (e.g., pH, enzymes) in different physiological environments, facilitating controlled drug release and protecting antigens and adjuvants from degradation by internal and external environments, thus improving vaccine stability and therapeutic efficacy [14]. Additionally, cancer nanovaccines can be used in conjunction with other therapeutic modalities, such as photothermal therapy and immune checkpoint inhibitors (ICIs), to achieve improved treatment outcomes [37].

## *Cancer* Nanovaccine Carriers

**Fig. 1:**
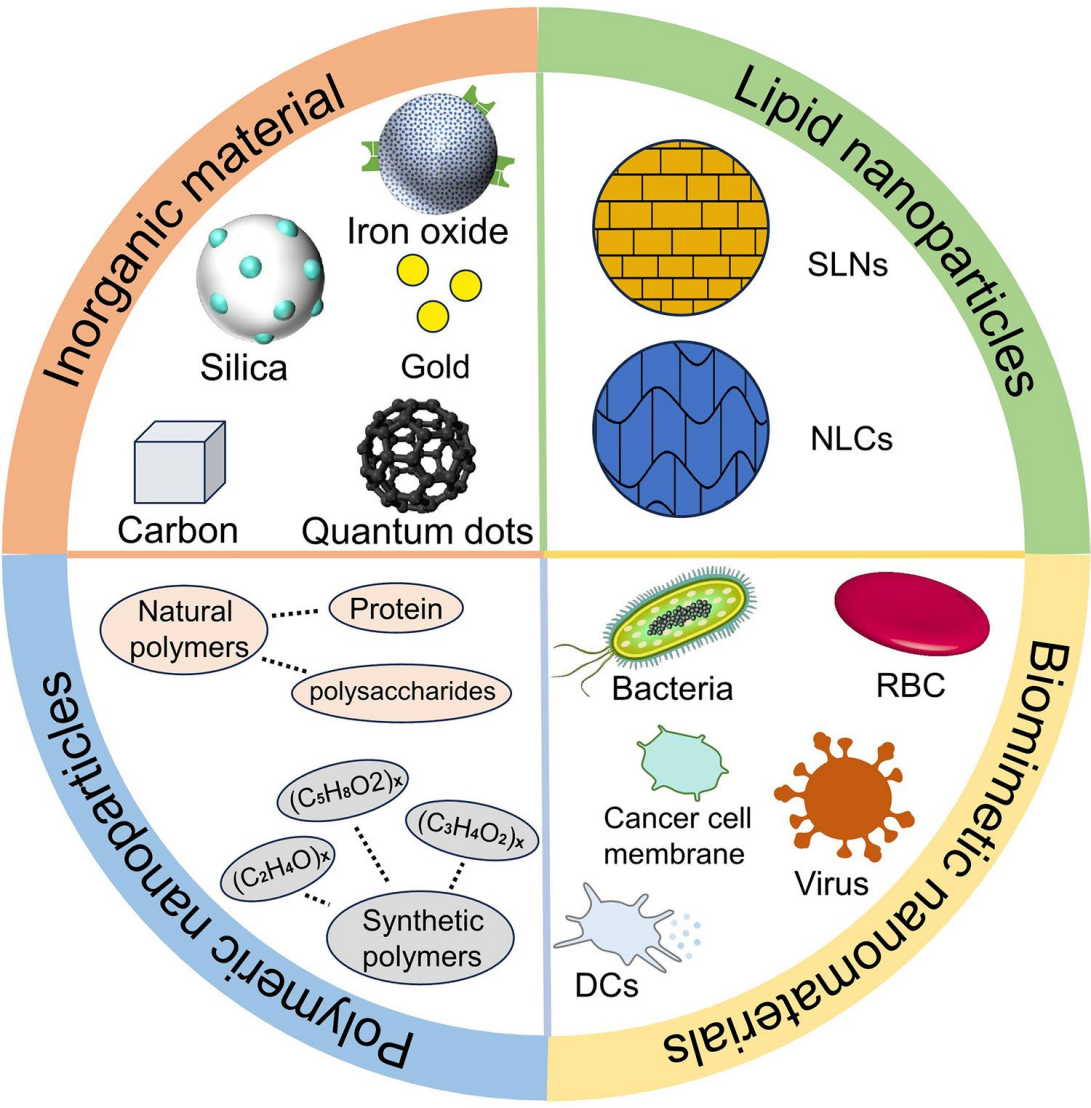
A summary on nanocarriers for engineering cancer nanovaccines

At present, cancer nanovaccine carriers are grouped into four primary classifications: inorganic nanoparticles, lipid-based nanoparticles, polymer-based nanoparticles, and biomimetic nanoparticles (Fig. 1). Different carriers have their own strengths and weaknesses, which will be examined sequentially.

### Inorganic Nanomaterials

Currently, in vaccine preparation, various inorganic substances are employed as carriers including gold [38], iron oxide [39], silica [40], quantum dots [41], and carbon nanomaterials [42]. These materials typically exhibit low biodegradability and maintain relative stability in vivo. Numerous inorganic nanoformulations have natural adjuvant properties [43] and are capable of creating multiple antigen structures [44], which help activate the immune system and improve the effectiveness of vaccines. Furthermore, in order to guarantee that vaccines are suitable for the living environment, it is essential to adjust the physicochemical characteristics of inorganic materials to improve biocompatibility [14]. Antigen substances and additional adjuvants are often modified on the surfaces of inorganic nanoparticles through chemical bonding and physical adsorption. For example, gold nanoparticles can be conjugated with CpG oligonucleotides (Toll-Like receptors 9 (TLR9) agonists) and tumor antigens, iron oxide nanoparticles (IONPs) can bind to tumor antigens and adjuvants such as MPL (monophosphoryl lipid A), silicon nanoparticles (SiNPs) can be combined with tumor antigens and adjuvants such as Poly(I) (TLR3 agonists), and carbon nanotubes (CNTs) can associate with tumor antigens and adjuvants like R837 (TLR7 agonists). The size of cancer nanovaccines delivered by inorganic nanoparticles typically favors the presentation by antigen-presenting cells (APCs), facilitating more robust immune uptake for the delivery of antigens and adjuvants [45]. Descriptions regarding gold and silica can be found in existing reviews [14], while here we focus on iron, quantum dots, carbon nanoparticles and carbonate nanoparticles.

Iron oxide nanoparticles exhibit high biocompatibility and superparamagnetism, enabling their controlled positioning and aggregation under external magnetic fields, thereby enabling targeted and controllable vaccine release to enhance vaccine efficacy and safety [39, 46]. Iron oxide also acts as an effective vaccine adjuvant to promote the polarization of proinflammatory macrophages [47], enhance immune cell activation, and stimulate cytokine production. Excessive generation of reactive oxygen species (ROS) can lead to biotoxic effects of iron oxide nanoparticles on DNA, proteins, and lipids in cell membranes [48], potentially harming healthy cells.

Quantum dots, which are semiconductor materials with distinct electronic configurations, have the ability to be adjusted in terms of their optical and electrical characteristics [41, 49]. Biological markers can be attached to biomolecules like peptides, antibodies, and nucleic acids through covalent bonds [50]. Quantum dots created through synthesis exhibit excellent fluorescence quantum efficiencies, stability against light exposure, and strong compatibility with living organisms, enabling their use in monitoring the spatial and temporal changes of vaccines over extended periods, as well as in identifying the specific locations of various tumors. For instance, recent studies have achieved real-time imaging of lymphatic flow in mice [41]. Nevertheless, the inclusion of highly toxic heavy metals, such as Cd, Se, and Te in quantum dots may be released within cells, inducing oxidative stress responses and thereby conferring a certain level of biotoxicity [51].

**Fig. 2:**
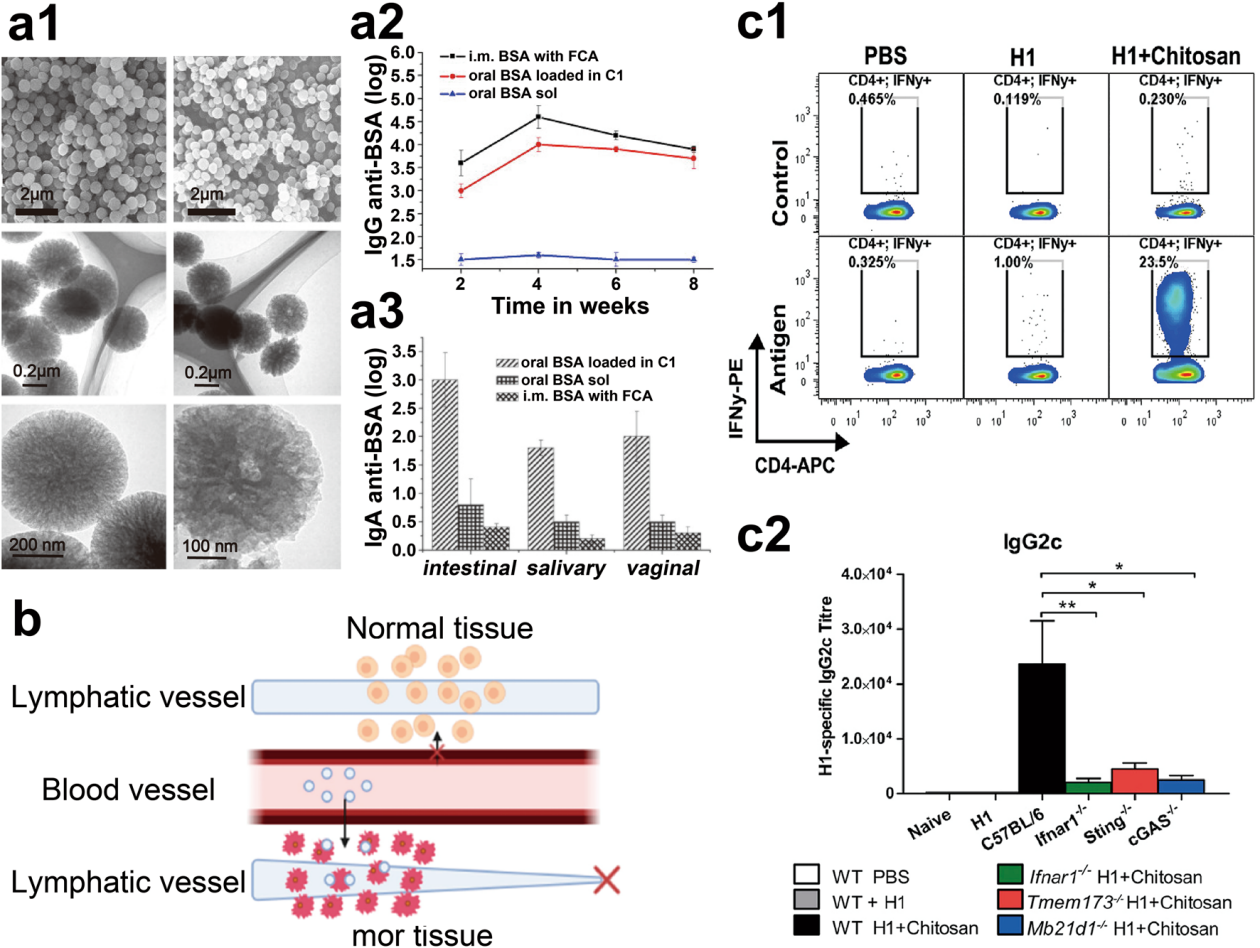
**a1** Transmission electron microscope images of novel hydrophobic carbon nanoparticles. **a2** Administering free BSA orally hardly induces the production of IgG, whereas loading BSA onto C1 for oral administration resulted in significantly elevated levels of immune response. **a3** Oral immunization with BSA loaded in carbon adjuvant can induce both systemic and mucosal immune responses. Reproduced with permission [42]. Copyright 2011, Elsevier. **b** Passive delivery mechanism of nanovaccines, wherein nanoparticles are firstly extravasated from the arteries, then diffused into cancerous tissues, and finally interact with intracellular or extracellular targets within the tumor microenvironment [61]. Copyright 2023, D. Sivadasan et al. **c1** IFN is crucial for chitosan-driven cellular immunity. **c2** Chitosan drives cellular immunity through the cGAS-STING pathway. Reproduced with permission [74]. Copyright 2023, Elsevier

Carbon nanoparticles have garnered significant attention as nanovaccine carriers [42, 52]. A new carbon nanoparticle that repels water, created by using silica as a model and sucrose as a source of carbon, has a diameter of 470 nm and pores measuring 40–60 nm, enabling it to hold a large quantity of antigens. Moreover, the carbon nanoparticles have a strong structure that can endure extreme conditions like those found in the stomach and intestines, which makes them ideal for use as oral vaccine enhancers (Fig. 2a1–a3). Their hydrophobic properties further facilitate uptake by M cells [42]. Nevertheless, a few research studies [53, 54] have suggested that carbon nanomaterials can also display specific harmful effects on living organisms, possibly causing the buildup of nanoparticles on the surface and inside the nucleus of cells, where they can interact with DNA and ultimately cause alterations in the production of proteins.

In carbonate nanoparticles, calcium carbonate nanoparticles are considered to be an effective carrier. By coating calcium carbonate nanoparticles with cancer cell membranes as carriers for Dox and Ce6, they can effectively serve as a DC vaccine for the treatment of breast cancer [55]. Simultaneously, manganese carbonate microspheres doped with calcium can be combined with perforin-Listeria monocytogenes hemolysin (LLO) as a vaccine delivery system for tumor immunotherapy [56].

### Lipid Nanoparticles (LNPs)

LNPs are composed of four main components: ionizable lipids, phospholipids, cholesterol, and polyethylene glycol-lipids (PEG-lipids). Among these, ionizable lipids are the primary component of LNPs. Phospholipids and cholesterol help maintain the structural integrity of LNPs, regulate the fluidity of the nanoparticles, and enhance the permeability of hydrophobic drugs [57, 58]. Due to their biodegradability, these lipid-based carrier systems are considered to be low-toxicity and safe nanocarriers [59, 60]. LNPs primarily induce enhanced immune responses and deliver antigens and adjuvants by effectively protecting antigens, increasing the size of antigen particles, and promoting endosomal escape of the antigens [58]. Depending on their composition, LNPs are classified into solid lipid nanoparticles (SLNs) and nanostructured lipid carriers (NLCs).

SLNs are small colloidal particles that range in size from 10 to 1000 nm and demonstrate strong physical stability. By altering the surface or structure of SLNs, for example, by adding functional groups, they can be designed to respond to particular environmental factors like pH, temperature, and ionic strength [61]. Furthermore, SLNs provide benefits like compact dimensions, extensive surface coverage, affordability, simple production process, and lack of toxicity [62]. Depending on the tumor environment and drug characteristics, SLNs can release nanoparticles passively (Fig. 2b), actively, or through synergistic mechanisms [61]. Nevertheless, SLNs exhibit a modest drug-loading capacity, and the crystallization process during storage could result in drug expulsion [63]. Generally, there are three methods in terms of incorporating antigens and adjuvants into SLNs: (1) uniformly distributing antigens and adjuvants within the SLN matrix (homogeneous matrix), (2) concentrating antigens and adjuvants-encapsulated lipid matrix within the SLN particles to generate a core-shell structure (enriched core), and (3) concentrating antigens and adjuvants on the surface of the SLNs (enriched coating) [64].

NLCs, which are lipid-based nanocarriers, are the next iteration of SLNs. Compared to the purely solid-state structure of SLNs, NLCs combine solid and liquid components to form carriers with a larger amorphous or partially crystalline matrix structure [65]. Consequently, NLCs exhibit higher drug-loading capacity and a highly controllable microstructure, and they can prevent drug release during storage by avoiding the presence of liquid lipids. At present, the manufacturing techniques for NLCs are divided into high-energy approaches like high-pressure homogenization and high-shear homogenization, low-energy methods including microemulsion, solvent diffusion, phase inversion, and membrane emulsification, and nearly energy-free methods like emulsification-solvent evaporation, emulsification-solvent diffusion, and solvent injection [66].

### Polymeric Nanoparticles

Polymeric nanoparticles are colloidal systems within the nanoscale range composed of polymer materials [67]. These nanoparticles exhibit excellent controllability and biocompatibility, making them suitable for targeted delivery of drug molecules. Through various modifications with charged molecules [68, 69], these nanoparticles can achieve targeted vaccine delivery. Positively charged nanoparticles, for example, can be taken up more effectively by APCs and absorbed by DCs [70]. In contrast, negatively charged polymer particles show low cellular uptake because of repulsion effects, which makes them better suited for delivering immune stimulants at the injection site [71]. Polymeric nanoparticles can be categorized into natural polymers (such as proteins and polysaccharides) or synthetic polymers (such as polyvinyl alcohol, polylactic acid, and polymethyl methacrylate).

Natural polymers possess excellent biodegradability and low cost. In vaccine delivery, the most commonly used natural polymer particles are chitosan. Chitosan carries a significant positive charge, enabling it to form tight complexes with anionic nucleic acids through electrostatic interactions [72, 73]. Its bioadhesive properties allow prolonged contact with mucosal surfaces, thereby promoting sustained antigen stimulation of immune cells. Recent studies have shown that chitosan can activate the DNA sensing system through the cGAS-STING pathway, inducing the production of type I interferons and promoting CD8 + T cell immune responses, which is beneficial for inducing cancer cell killing (Fig. 2c1, c2) [74]. In other studies, an example of this is hyaluronic acid, which is made up of glucuronic acid and N-acetylglucosamine units connected by β-1,4 and β-1,3 glycosidic bonds and can serve as a drug delivery system (DDS) by attaching to the CD44 receptor found on the exterior of tumor ECM [75]. Another example is the negatively charged nanovaccine shell composed of ovalbumin and hyaluronic acid, which exhibits highly immunostimulatory properties and storage stability, suitable for activating immune cells within the skin [76]. In peptides carriers, inducing self-assembling peptides to form nanoparticles of 20–200 nm as carriers for the development of cancer vaccines can trigger a stronger T-cell immune response and anti-tumor effects. Researchers have developed a peptide-based HPV vaccine using nanoparticle technology [77].

Synthetic polymers demonstrate exceptional flexibility due to their tunable structures and drug-loading capacities. Poly(lactic-co-glycolic acid) (PLGA) synthetic polymer particles generally exhibit higher reproducibility and easier control over the slow release of antigens compared to natural polymer particles. For example, Koerner et al. designed PLGA particles encapsulating antigens and double-stranded RNA (dsRNA) adjuvants, successfully increasing the number of targeted lymph nodes and effectively being taken up and presented by DCs, ultimately producing effective and long-lasting antitumor immune responses [45]. Moreover, the dopamine polymer carrier, formed by polymerizing dopamine on the surface of PLGA nanoparticles, was demonstrated to stimulate Toll-like receptor 9 pathways in mice studies, ultimately boosting the immune response [78]. This discovery provides a new perspective for cancer treatment research. In cationic polymers, cationic polymer-lipid hybrid nanovesicle (P/LNV)-based liposomes have been developed to deliver tumor vaccines to enhance the immunogenicity of peptide antigens and block immune checkpoints for the treatment of melanoma [79]. Gao et al. developed a conjugated diradical polymer nanoparticle, TTB-2 nanoparticles, which achieved effective tumor photothermal ablation in the near-infrared (NIR)-II window through photoacoustic imaging guidance with no significant side effects. In addition to tumor eradication, this study also demonstrated that the efficient photothermal effect could prevent lung metastasis of breast cancer [80].

Despite the high biodegradability exhibited by polymeric nanomaterials, their potential biotoxicity cannot be overlooked. Therefore, before clinical application, it is imperative to strictly control the dosage and ensure their metabolism to prevent severe toxic effects on the human body [81].

### Biomimetic Nanomaterials

The design of biomimetic nanomaterials is inspired by the structure and functionality of biological organisms, allowing these materials to replicate specific biological properties and functions [82]. By mimicking cellular structures, nanomaterials can enhance the biocompatibility of vaccines and perform functions similar to those of cells. Biomimetic nanomaterials possess distinctive advantages. Their shape, size, and surface characteristics enable them to deliver water-insoluble drugs, control drug release, and improve pharmacokinetics, ensuring efficient drug diffusion within the body. These small-sized particles can traverse the narrowest capillaries, thereby passively targeting tumors. Passive targeting through enhanced permeability and active targeting via ligands interacting with specific cell surface receptors are the two primary strategies for biomimetic nanomaterials to target tumors. Their formulations typically include integrins involved in cell adhesion, angiogenesis, and solid tumor metastasis. Additionally, loading other nanoparticles can enhance the vaccine’s cytotoxicity and targeting capability, such as loading palladium nanoparticles for photothermal ablation and imaging [83]. Current research is exploring the potential applications of these technologies in mimicking eukaryotic cells, bacteria, and viruses [84].

In studies involving eukaryotic cells, cell membrane-coated nanomaterials utilize cell surface receptors for specific biomolecular recognition. Typically, PLGA nanoparticles encapsulated by red blood cell membrane and containing paclitaxel (PTX) and tumor-penetrating peptide IRGD have been employed in the management of metastatic breast cancer [85]. Another study utilized magnetic mesoporous silica nanoparticles (MMSNs) coated with red blood cell membranes (RBC) to form RBC@MMSNs, achieving high nanoparticle accumulation in tumors under magnetic field induction. Upon exposure to light, singlet oxygen is rapidly generated, leading to tumor tissue necrosis. This nanovaccine effectively integrates immunotherapy with photodynamic therapy, providing an innovative strategy for cancer treatment [86]. Li et al. developed an artificial red blood cell (FTP@RBCM) based on Fe-porphyrin frameworks (FTPs) capable of generating a high abundance of free radicals for tumor therapy. FTP@RBCM can accumulate significantly at tumor sites to induce tumor cell death. Moreover, it triggers a robust systemic anti-tumor response when combined with T-cell immunoglobulin and mucin domain 3 (Tim-3) checkpoint blockade. This biomimetic red blood cell membrane offers a novel direction for tumor treatment [87].

Cell modifications alone are sufficient to achieve the immunostimulatory effects of vaccines [88, 89]. DCs-originated exosomes (DEX) specifically contain immune-stimulating components found in mature DCs, like peptide-major histocompatibility complex (p-MHC) and CD86 co-stimulatory molecules, essential for activating T cells internally [89]. Modifying αCD3 and αEGFR on DEX enables dual-specificity binding to T cell surface CD3 and cancer cell surface epidermal growth factor receptor (EGFR), promoting interaction between T cells and cancer cells. This design of dual-specificity DEX demonstrates its potential to inhibit tumor recurrence and metastasis [90].

In the field of bacterial applications, researchers have developed strategies utilizing bacteria as antigen carriers. For instance, coating *Escherichia coli* surfaces with lipid nanoparticles containing photosensitizers enhances their invasive capabilities against cancer cells and achieves efficient photodynamic therapy [91]. Furthermore, a new DNA vaccine has been developed through the encoding of plasmid DNA with vascular endothelial growth factor receptor 2 (VEGFR2) and particular antigens. DNA vaccines are created by combining β-cyclodextrin-polyethyleneimine (PEI) and plasmid DNA (pDNA) through electrostatic self-assembly, then attaching them to the surfaces of invasive Salmonella bacteria to aid in their transportation [92, 93]. The use of bacterial outer membrane vesicles (OMVs) has become a valuable method for delivering drugs because of their strong compatibility with living organisms, their ability to hold a lot of drugs, and stable physical and chemical properties [94]. The abundant pathogen-associated molecular patterns (PAMPs) within OMVs confer them with high immunogenicity, which makes them capable of attracting and activating immune cells at tumor sites. Studies have shown that intravenous injection of Escherichia coli-derived extracellular vesicles (EVs) can effectively eradicate diverse types of cancers, including colorectal cancer, metastatic breast cancer, and metastatic melanoma [95].

Virus-like particles (VLPs) are tiny particles created through the self-organization of proteins found in viruses, able to present particular antigens on their exterior to trigger immune responses from both antibodies and cells [96, 97]. For example, loading HPV16L2 protein onto MS2 VLPs produces L2-MS2 VLPs that can induce high titers of anti-L2 IgG antibodies. Vaccination with these VLPs effectively protects mice from infection with HPV pseudoviruses PsV31 and PsV45, demonstrating their potential application in preventing human papillomavirus infection [98]. Furthermore, research has indicated that incorporating the abundantly produced HBV X protein onto VLPs can stimulate a heightened number of particular CD8 + T cells, leading to a more robust immune reaction in comparison to a solitary peptide [99]. Virosomes, engineered virus-like nanoparticles consisting of viral envelope fusion proteins and other membrane proteins, serve as scalable vaccine carriers [100]. Her2/neu peptides can be attached to influenza virosomes in breast cancer therapy to stimulate immune reactions against tumor cells that have an excessive amount of Her2/neu [101]. Meanwhile, virus-mimetic nanoparticles, resembling the shape and size of viruses, also show potential as vaccine carriers. Studies have shown that melanoma vaccines prepared using this method can delay disease progression [84].

Despite the considerable potential demonstrated by the aforementioned nano-material carriers for drug loading and delivery, several deficiencies hinder their widespread application. Inorganic material carriers and polymer nanomaterials, while relatively easy to fabricate, exhibit significant biotoxicity [48, 81]. The lipid nanomaterials have good biocompatibility, but there may be drug leakage and low encapsulation efficiency leading to reduced stability [102]. Biomimetic nanomaterials, though exhibiting the strongest immunogenicity, face challenges related to their complex fabrication processes and the excessive toxicity of certain pathogen-like carriers [82]. Future research should prioritize enhancing the fabrication of nanotechnologies, reducing production complexity, and ensuring biosafety while improving immunogenicity and stability.

### Comparisons Among Different Nanovaccines Carriers

Cancer nanovaccines encompass a wide variety of carriers. Selecting appropriate carriers as a focus for future research and clinical applications can expedite the clinical translation of cancer nanovaccines. Comprehensive comparative analysis of various carriers in terms of their effectiveness, cost, ease of production, and scalability may assist researchers and clinicians in making informed decisions.

Inorganic nanomaterials possess unique electrical, optical, and magnetic properties, making them suitable for multimodal imaging and therapy. For instance, iron oxide nanoparticles can be used for magnetic resonance imaging (MRI) and can also be guided by an external magnetic field for targeted therapy. Additionally, inorganic nanomaterials are typically more stable and less prone to degradation compared to organic materials. This stability allows them to maintain activity for extended periods in vivo and accumulate in tumor sites via the enhanced permeability and retention (EPR) effect, thereby improving drug delivery efficiency [103]. However, inorganic nanomaterials pose significant toxicity concerns due to their difficulty in degradation and clearance from the body, potentially leading to inflammatory responses or fibrosis. The synthesis and functionalization of inorganic nanomaterials are often complex, requiring precise control over size, shape, and surface properties. This precision in production incurs high costs, particularly when scaling up for large-scale production [104, 105].

Lipid nanomaterials can effectively deliver antigens and adjuvants to target cells, particularly accumulating in tumor sites via the EPR effect. As endogenous components, lipid nanoparticles can protect antigens from enzymatic degradation in the body, enhancing the stability and efficacy of vaccines and reducing immune system rejection. Furthermore, after specific modifications, they can bind to various drugs and antigens, offering the potential for multimodal therapy and imaging. However, studies have shown that lipid nanoparticles with good biocompatibility can sometimes induce immune reactions, particularly when modified with polyethylene glycol (PEG), which can activate the production of anti-PEG antibodies and lead to CD8 + T cell infiltration [106]. mRNA vaccines using lipid nanomaterials as carriers require ultra-low temperature storage and transportation, increasing logistical and usage complexity [107–109]. The preparation and functionalization processes of lipid nanoparticles are also complex, requiring precise control over size, shape, and surface properties [110]. Nonetheless, due to the successful development and clinical application of lipid-based mRNA vaccines during the COVID-19 pandemic, the technology is relatively mature, and costs are lower. Among various nanomaterial carriers, lipid nanoparticles have advantages in production and scalability.

For polymeric nanomaterials, their chemical structures and physical properties can be optimized by adjusting synthesis conditions and formulations, allowing precise control over particle size, shape, and surface characteristics, thereby enhancing the stability and targeting of the vaccine. Additionally, they possess good biocompatibility and can be engineered with controlled-release functions to gradually deliver antigens or drugs, thereby improving the vaccine's persistence and therapeutic efficacy [111]. Currently, peptide-based cancer nanovaccines possess considerable potential. However, they still present numerous side effects. For instance, the injection of new antigens might lead to genomic alterations within the tumor, thereby triggering endogenous T cell immune responses. Furthermore, abnormal gene expression within the tumor may cause high-affinity T cell receptors (TCRs) to be occupied, resulting in severe side effects [112, 113]. To achieve more robust anti-tumor responses and reduce immune evasion, new tumor-specific antigens (TSAs) or tumor-associated antigens (TAAs) need to be rapidly and efficiently screened out and manufactured. Currently, direct acquisition from patients offers extremely high individual specificity, making widespread application difficult. Conversely, predictive reverse immunology can simulate and construct a broad range of undiscovered epitopes, providing both high specificity and broad applicability. However, due to the complexity of MHC-II restricted peptides compared to MHC-I, predicting and identifying peptides that bind to MHC-II molecules and assist T cells remains very challenging [114].

For biomimetic materials, they typically mimic the structure and function of natural biological materials, resulting in excellent biocompatibility. These materials can effectively protect antigens in vaccines from degradation by enzymes or other mechanisms in the body, enhancing the stability and persistence of the antigens while reducing immune reactions and toxicity. Similar to lipid nanomaterials, biomimetic nanomaterials can be surface-modified and functionalized to specifically target cancer cells or the tumor microenvironment (TME), increasing the accuracy and efficiency of drug delivery. Biomimetic nanomaterials can incorporate multiple functions, such as carrying various antigens, regulating release rates, and providing imaging guidance, making them a versatile platform that enhances the overall efficacy of vaccines [83]. However, the design and manufacturing process of biomimetic nanomaterials is often complex, involving multiple steps and high-precision technologies like molecular self-assembly and nanoimprinting. This requires sophisticated equipment, resulting in high production costs and significant preparation challenges. Additionally, due to technical and cost limitations, the scalability of these materials is currently not promising, primarily meeting laboratory research needs and making large-scale and consistent product manufacturing difficult [115–117].

## Design Principles and Action Targets

### Cellular Immunity-Targeting DCs

**Fig. 3:**
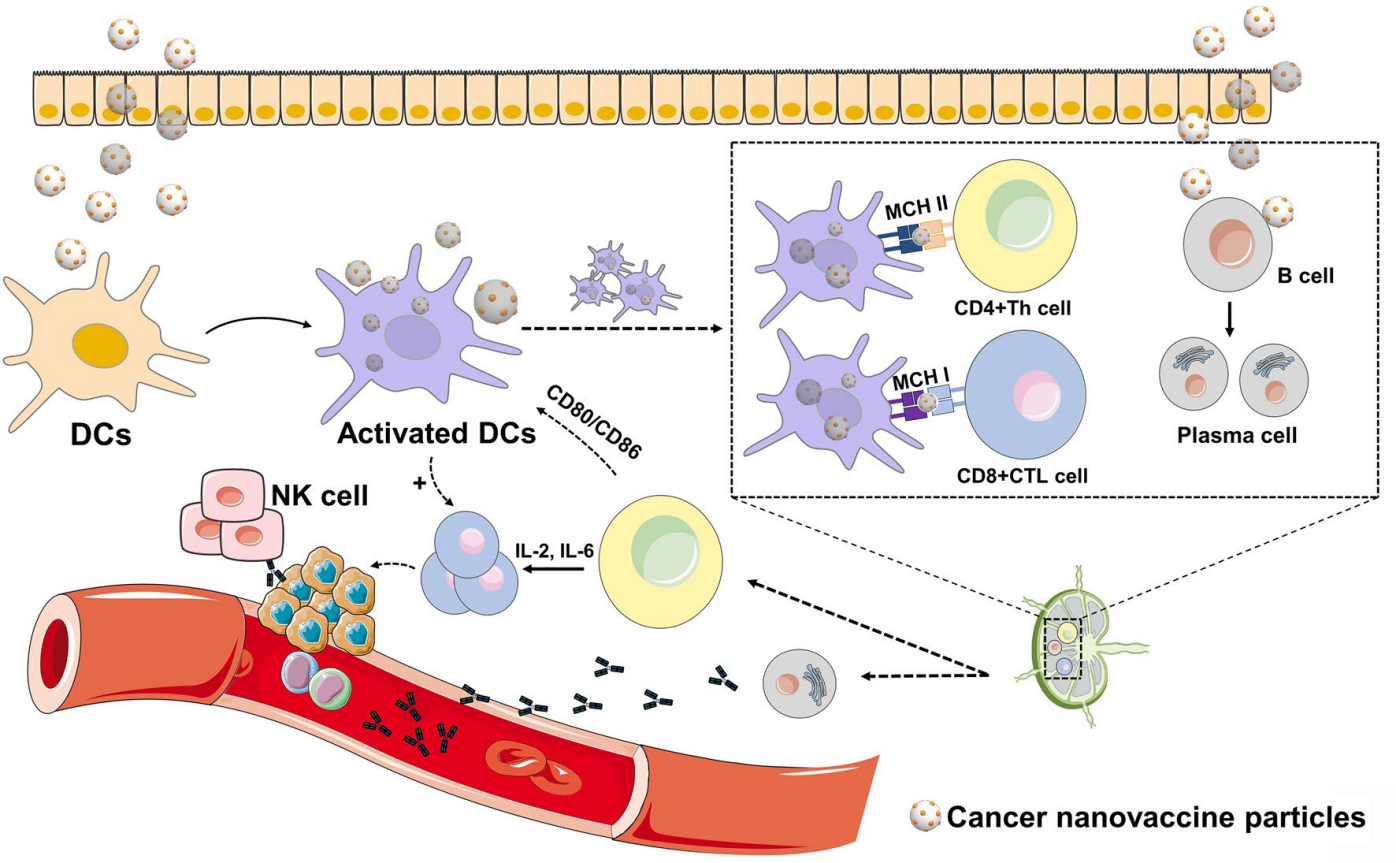
Schematic of the mechanism by which cancer nanovaccines target DCs and activate immune responses in the body. Upon entry into the body, the nanoparticles are taken up by DCs and other antigen-presenting cells. Subsequently, the antigens are presented to T cells within the lymph nodes, promoting T cell differentiation and maturation. This process also activates B cell-mediated immune responses. The mature immune cells then exit the lymph nodes and migrate to the tumor invasion area to exert their cytotoxic effects

Cellular immunity plays a central role in anti-tumor responses. DCs, as the most efficient APCs, capture tumor antigens and display them to T cells using MHC molecules, initiating T cell-mediated tumor destruction [118]. Immature DCs originate from progenitor cells in the bone marrow. Through surface pattern recognition receptors (PRRs), such as Toll-Like receptors (TLRs) and C-type lectin receptors (CLRs), immature DCs recognize damage-associated molecular patterns (DAMPs) or PAMPs in their surrounding environment. This allows DCs to uptake antigens via mechanisms such as phagocytosis and receptor-mediated endocytosis. After encountering and capturing antigens in various tissues and organs, or upon exposure to certain inflammatory stimuli (e.g., LPS, IL-1β, TNF-α), DCs express specific chemokine receptors and migrate to systemic lymphoid and non-lymphoid tissues in response to chemokine signaling. Throughout this migration, immature DCs progressively differentiate into mature DCs through the action of multiple transcription factors, signaling molecules, growth factors, cytokines, chemokines, and adhesion receptors. During migration, DCs continuously process antigens and express costimulatory molecules. Upon reaching peripheral immune organs, they mature into fully developed DCs. Based on microenvironmental signals, they alter the expression of chemokine receptors and adhesion molecules on their surface. In response to chemotactic signals, they migrate into secondary lymphoid organs, where they present the processed antigens to T cells (Fig. 3) [119–121]. Additionally, DCs further enhance immune responses by secreting cytokines such as interferon-gamma (IFN-γ) and interleukins (ILs) to communicate with T cells. Cancer nanovaccines achieve cellular immunity by targeting various receptors on immature DCs, enabling antigen uptake, processing, and presentation. Currently, there are two main targeting strategies: one is passive targeting of relevant DCs, where cancer nanovaccines are injected into areas where DCs aggregate, such as skin injection, to quickly reach lymph node clusters; the other is active targeting of DCs, where vaccine antigens are delivered directly to resident DCs by coupling with monoclonal antibodies (mAbs) specific to DC surface receptors [122, 123]. However, passive targeting of DCs lacks specificity, resulting in lower immune effects, which is not conducive to the development of cancer nanovaccines. Therefore, current research efforts are mainly focused on actively targeted dendritic cell vaccines.

#### CLRs Family

Utilizing CLRs found on DCs is a successful approach for vaccine development. CLRs can identify the polysaccharide components on tumor cells or pathogens through carbohydrate recognition domains [124], which helps in antigen presentation. Antibodies targeting surface receptors of DCs such as DEC205 (CD205), Langerin, CLEC9A, Mincle, Mannose receptor (MR), etc., have been developed [125–127]. Utilizing nanoparticles modified with the aforementioned antibodies may be advantageous for cancer vaccine preparation.

**Fig. 4:**
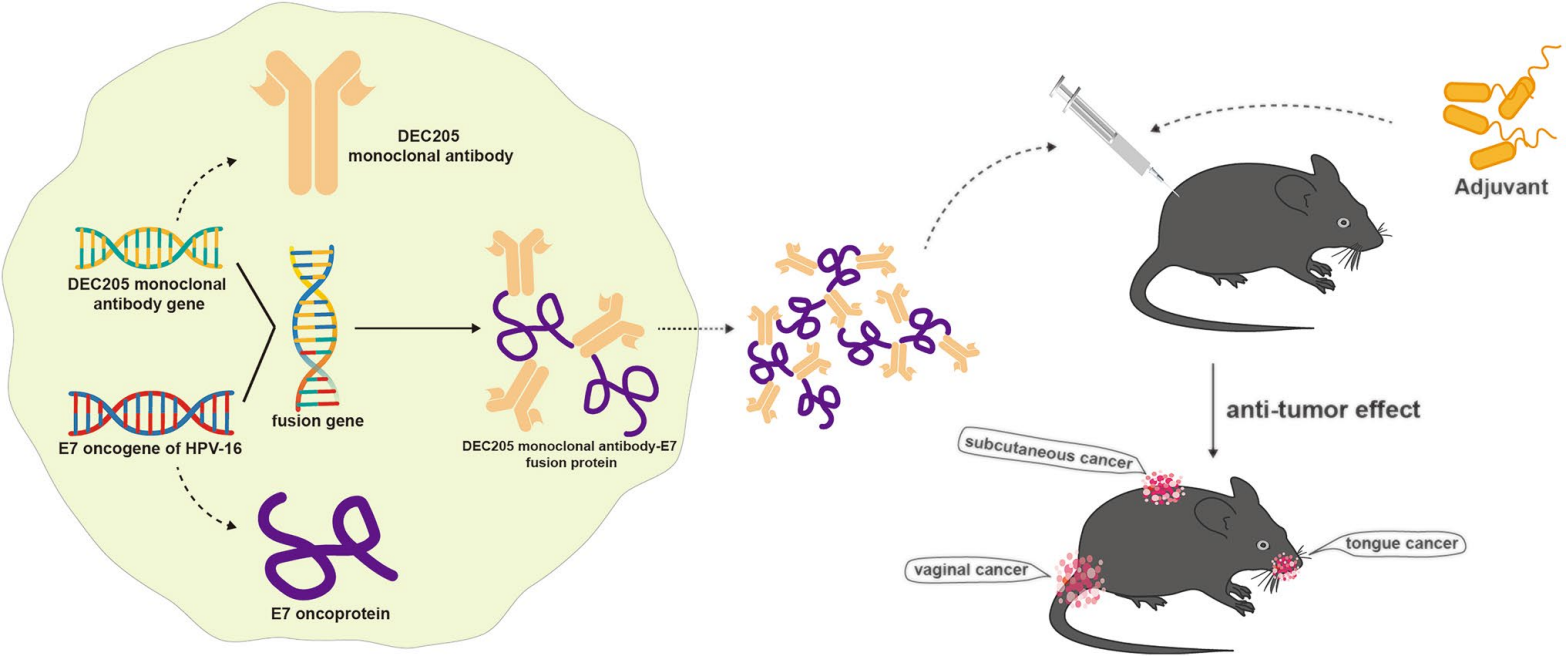
Fusion of DEC205 monoclonal antibody gene with HPV16 E7 oncogenic protein gene to generate a combined vaccine. Concurrent use with immune adjuvants elicits potent anti-tumor effects on subcutaneous, tongue-side, and vaginal tumors

DEC205 is a member of the macrophage MR family and acts as a receptor for type B oligonucleotides. High levels of this gene are found in conventional dendritic cell 1 (cDC1) and it is also present in a few other cell types [128–130]. In one instance, researchers combined anti-DEC205 monoclonal antibodies with the HPV-16 E7 oncogenic protein to develop a therapeutic vaccine aimed at treating HPV-related tumors. After combining with an adjuvant, αDEC205-E7 mAb can activate CD8 + T cells that target tumor antigens in both systemic and lymphoid tissues (Fig. 4), resulting in strong anti-cancer responses in different tumor models [131]. Another study found that modifying the Fc portion of DEC205 monoclonal antibodies can enhance their binding to FcRn, leading to an extension in the duration and effectiveness of the vaccine [123]. Phung et al. developed NVs containing an artificial tumor membrane (IMQ/siR@ATM-NVs) that target DEC205 receptors which can block IL-10 secretion so that enhance the activation of co-stimulatory molecules and the secretion of Th1 cell cytokines, facilitating the infiltration of cytotoxic T lymphocytes (CTLs) and natural killer cells (NK cells) into tumor sites [132].

Langerin receptor is a kind of membrane protein expressed by Langerhans cells, a subset of human skin DCs [133–135]. In a research project, a fusion protein was created by cloning that merged anti-human Langerin antibody with Epstein-Barr virus nuclear antigen 1 (EBNA1). Stimulation with EBNA1 peptide led to elevated levels of IFN-γ- and TNF-α-positive CD4 + T cells, indicating the potential of Langerin-targeted vaccines in fighting tumors. However, unfortunately, the immune response elicited by Langerin-EBNA1 in skin implants was not significant, possibly due to the limited migration of Langerhans cells [136].

**Fig. 5:**
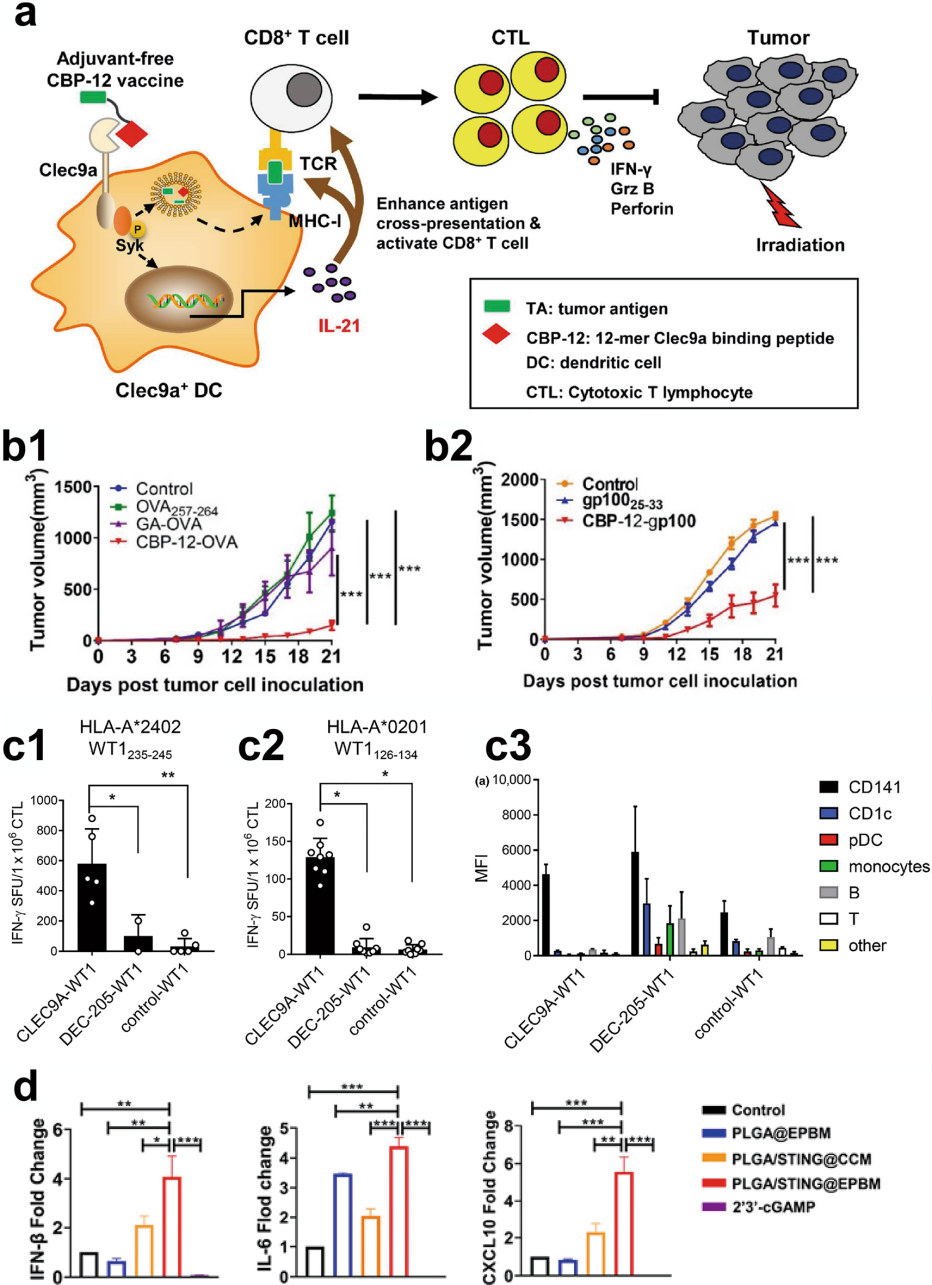
**a** Proposed model by which the adjuvant-free CBP-12 conjugated peptide vaccine elicits an IL-21 dependent antitumor response by targeting Clec9a on DCs. **b1** Following CBP-12-OVA treatment, the tumor volume in mice was significantly reduced. **b2** Following CBP-12-OVA treatment, the tumor volume in mice was significantly reduced. Reproduced with permission [138]. Copyright 2021, S. Gou et al. **c1**, **c2** When cross-presentation was performed using WT1 epitopes 235-245 and 126-134, CLEC9A-WT1 demonstrated a significant advantage. **c3** Although DEC-205-WT1 can target a larger number of cells, CLEC9A-WT1, through cross-presentation, can achieve the same effect by targeting only CD141. Reproduced with permission [139]. Copyright 2020, F. E. Pearson et al. **d** After treatment of Clec9a DCs with PLGA/STING@EPBM (engineered peptide), the expression of key mediators for anti-tumor T cell activation and recruitment, namely IFN-β, IL-6, and CXCL10, was significantly increased. Reproduced with permission [143]. Copyright 2021, American Chem. Society

CLEC9A, also referred to as CD141, is a member of the V group of C-type lectin-like receptors (CTLRs) and functions as a stimulatory receptor, facilitating the presentation of antigens associated with dead cells in a manner dependent on Syk [137]. A study developed a 12-mer peptide carrier (CBP-12) with a high affinity for Clec9a, intended for use as a vaccine carrier. This peptide can stimulate Clec9a + DCs to produce IL-21 while reducing the neutralizing antibody blockade by activating Syk without inducing IL-12 (Fig. 5a, b1 and b2) [138]. Another attractive vaccine fuses Wilms' tumor 1 (WT1) with a human anti-CLEC9A antibody, specifically delivering WT1 to CD141 + DCs. This strategy indicates higher therapeutic efficacy against WT1-expressing cancers such as acute myeloid leukemia (AML) (Fig. 5c1–c3) [139]. Additionally, a nanovaccine targeting CD141 + DCs loaded with tumor antigen Melan-A and α-galactosylceramide (a potent activator of invariant natural killer T (iNKT) cells) was designed. The nanovaccines lead to the activation of CD8α + DCs and iNKT cells in humanized mice, which also shows iNKT cells can activate DCs in the body [140]. Moreover, STING agonists markedly enhance the production of type I interferon (IFN) in Clec9a + DCs [141, 142]. Using this information, a group of researchers developed a nanovaccine delivery system (PLGA/STING@EPBM) that is covered with a biomimetic cancer cell membrane expressing an EPBM. The nanovaccine boosts IFN-stimulated gene expression and enhances antigen cross-presentation, leading to the suppression of melanoma and breast tumor growth (Fig. 5d) [143].

Mincle, alternatively referred to as Clec4e or Clecsf9, its activation triggers the FcRγ-Syk-Card9-Bcl10-Malt1 signaling pathway and then triggers immune responses by involving Th1/Th17 immune cells [144]. Studies have demonstrated that utilizing Mincle agonists as carrier molecules and intrinsic adjuvants, coupling STn (a glycan antigen used for cancer vaccines) with two Mincle agonists vizantin and TDB which successfully eradicated cancer cells in animal experiments [145]. Recent research has combined NOD with Mincle, using conjugated brartemicin and MDP ligands to simultaneously target NOD2 and Mincle PRRs, thereby enhancing the antitumor response [146]. The MR is a type of immune receptor that is found in abundance on the cell membrane of macrophages and DCs [147]. It possesses multiple extracellular domains that enable it to recognize and bind various endogenous and exogenous ligands [148]. For example, a study reported the development of nanoparticles incorporating a novel lipid-like mannoside mimic with bis-dioxopiperazine and guanidine structures. This design facilitates the effective delivery of DNA vaccines [149]. In another study, a novel nanoparticle was developed by conjugating mannose to a poly-L-lysine-riboflavin chain (PLL-RT) to form mannose-modified PLL-RT (Man-PLL-RT). The Man-PLL-RT-mediated nanovaccine significantly enhanced the endocytosis, maturation, and cross-presentation of DCs. When combined with PD-L1 blockade therapy, this approach markedly reduced tumor volume in a murine melanoma model [150].

CLRs are primarily expressed on DCs and are specialized in recognizing carbohydrate structures on antigens. These receptors are highly involved in capturing and presenting antigens to T cells, facilitating the presentation of antigens on MHC I and MHC II molecules, thus directly linking to the activation of CD4 + and CD8 + T cells. Additionally, CLRs can influence dendritic cell maturation and cytokine production, thereby modulating both innate and adaptive immunity. Some CLRs can promote cross-presentation, which involves presenting exogenous antigens via MHC I molecules to activate CD8 + T cells. These advantages are particularly important for antiviral and antitumor immune responses. Overall, targeting different CLRs on DCs as a strategy for developing cancer nanovaccines is a highly promising approach.

#### Scavenger Receptor Class B Type 1 (SR-B1) on DCs

SR-B1, a heavily glycosylated glycoprotein of type 3, aids in the absorption of cholesterol esters from high-density lipoprotein in the liver [151]. A team has designed a biocompatible nanovaccine (α-Ap-FNP) with a size of approximately 30 nm, which can effectively accumulate in draining lymph nodes. The vaccine utilizes both general and SR-B1-focused methods to transport antigen peptides (Ap) to migratory DCs for antigen presentation, offering a hopeful approach for efficient antigen presentation and robust T-cell activation for cancer immunotherapy [152]. Compared to C-type lectin receptors, which primarily recognize carbohydrate structures, SR-B1 can recognize and bind multiple ligands, including oxidized low-density lipoprotein, pathogens, and cellular debris [153]. This diversity allows DCs to process a wider range of antigens, enhancing the efficiency of antigen capture and uptake. Therefore, targeting SR-B1 for the development of cancer nanovaccines holds great potential in cancer therapy.

#### TLRs Family

TLRs are single, transmembrane, non-catalytic proteins involved in innate immunity, and they serve as a bridge between innate and adaptive immunity. TLRs expressed on DCs can recognize various PAMPs.

TLR2 is a membrane protein that plays a crucial role in the immune system. It is primarily expressed on the surface of cells such as macrophages and DCs, where it can recognize and bind PAMPs, including bacterial lipoproteins and yeast β-glucans [154]. Zhao et al. designed a cancer nanovaccine utilizing outer membrane vesicles (OMVs) as carriers, employing a Plug-and-Display system. This system allows OMVs to present a comprehensive antigenic profile of tumor cells and rapidly display it on the outer membrane surface. Subsequently, the vaccine induces an anti-tumor response by activating the TLR2/4/5 signaling pathways [155]. MPLA is the only component licensed for use in human vaccines that specifically targets TLR2 as an agonist [15]. Therefore, researchers have developed AS01, which is composed of liposomes and monophosphoryl lipid A (MPLA); AS02, which contains MPLA and QS-21 in a water-in-oil emulsion; and AS04, which includes MPLA and aluminum salts. These formulations are designed to enhance cellular immune responses and induce tumor cytotoxicity [156].

TLR3 is an important membrane protein expressed in DCs, macrophages, and other immune cells. It plays a crucial role in the innate immune system, wherein viral infections are primarily detected through recognizing double-stranded RNA (dsRNA). Once recognizing dsRNA, TLR3 activates signaling pathways that lead to the production of interferons and other cytokines, thereby initiating an antiviral immune response. Additionally, TLR3 can modulate the intensity and duration of inflammatory responses [157]. A novel strategy targeting TLR3 involves the use of synthetic double-stranded RNA (Poly I:C) to bind to TLR3. Due to the severe side effects associated with Poly I:C [158], many studies have modified it to reduce adverse reactions but still retain tumoricidal effects. Poly-ICLC is a modified form of Poly I:C stabilized with polylysine, which exhibits RNAse resistance. Research has shown that Poly-ICLC is effective against tumors such as malignant gliomas and anaplastic astrocytomas [159–162]. Other Studies have introduced modifications to Poly I:C by incorporating unpaired bases, such as uracil and guanine, to reduce its toxicity. Numerous investigations have confirmed that these modifications significantly decrease toxicity in vitro experiments, suggesting that modified Poly I:C holds promise as a novel TLR agonist [163, 164].

TLR4, or CD284, is a pattern recognition receptor primarily located on the surface of immune cells like macrophages and DCs, playing a key role in immune stimulation [165, 166]. Numerous nanoparticle enhancers that target TLR4 have been demonstrated to stimulate the production of inflammatory cytokines in DCs [167–169], potentially playing a vital role in eliminating cancer cells. Yang et al. created a completely artificial cancer vaccine (MPLA-Tn-KRN7000) using the Tn antigen, which is commonly found in cancer cells, along with the TLR4 ligand MPLA and the iNKT cell agonist KRN7000 to enhance its effectiveness. Comparative immunological research on wild-type and TLR4-deficient mice showed that MPLA-Tn-KRN7000 can trigger strong Tn-specific and T cell-mediated immune reactions, leading to combined stimulation of TLR4 and iNKT cells, highlighting the promise of MPLA-Tn-KRN7000 as a potential cancer immunization [170]. Parsons and colleagues utilized a lentiviral vector (ZVex®) that targets DCs, along with the TLR4 agonist G100, to treat melanoma and glioblastoma tumor mouse models. This combination therapy activated TLR4 on macrophages and DCs in the TME, leading to Th1-type inflammatory responses. As a result, 88.9% of mice experienced complete tumor regression, with long-term survival and no recurrence. This approach demonstrated the potential to induce tumor regression in murine tumor models [171]. Baljon et al. utilized a flexible limited jet mixing technique to co-encapsulate several peptide antigens with different physical characteristics and diverse vaccine enhancers into vesicular nanoparticles that respond to changes in pH. The researchers discovered that combining the complementary enhancers STING activator cGAMP and TLR4 activator monophosphoryl lipid A (MPLA) in nanocarriers had a synergistic effect on boosting dendritic cell co-stimulatory markers, releasing proinflammatory cytokines, and presenting peptide antigens, leading to higher levels of peptide antigens in lymph nodes and uptake by DCs in draining lymph nodes, ultimately triggering strong CD8 + T cell immune reactions. Ultimately, the cancer nanovaccine system enhanced the effectiveness of treatment in a mouse model of colon cancer [172]. Compared to CLRs, which regulate immune responses through non-inflammatory pathways for fine-tuning, activation of the TLR4 receptor induces the production of a large number of cytokines through the MyD88 and TRIF signaling pathways, significantly enhancing the activation and maturation of DCs [173]. Therefore, targeting TLR4 to stimulate DCs for secondary immune responses is a promising strategy for developing cancer nanovaccines.

TLR7 primarily detects viral infections by recognizing single-stranded RNA (ssRNA). This recognition activates signaling pathways that lead to the production of interferons and other cytokines. Recently, TLR7 has also emerged as a significant target for cancer nanovaccines [174]. Xia et al. designed a pH-/enzyme-responsive nanovaccine (TNV) that incorporates TLR7/8 agonists. This smart nanovaccine can intelligently respond to the endosomal environment, precisely releasing TLR7/8 agonists, and has demonstrated strong therapeutic efficacy against melanoma and colon cancer in mice [175]. Mo et al. developed a nanovaccine (R837/LNP-M-L) based on high-density lipoprotein (HDL)-mimicking nanoparticles, which encapsulates the TLR7/8 agonist R837 and is capable of efficiently targeting lymph nodes. This nanovaccine demonstrated effective tumor-killing against melanoma in mice [176]. The locally used TLR7 agonist Imiquimod has been approved by the FDA for the treatment of viral infections and skin cancers. Additionally, TLR7 agonists such as TQ-A3334, APR003, SHR2150, and RO7119929 have entered clinical trials and hold promise as effective therapeutic agents [177]. Zhang et al. synthesized multicomponent nanovaccines (MCNVs) composed of the STING agonist CDGSF and the TLR 7/8 agonist 522. This formulation elicits a broader cytokine response and enhances antigen cross-presentation by activating bone marrow-derived dendritic cells (BMDCs), thereby stimulating specific anti-tumor T cell responses. In in vivo experiments, MCNVs resulted in significant tumor shrinkage and a 100% survival rate, indicating their potential to improve the durability and efficacy of cancer immunotherapy [178]. Wang et al. designed redox-responsive antigen nanoparticles that covalently bind with imidazoquinoline-based TLR7/8 agonists. These nanoparticles are intended for lymph node-targeted immune activation, which can enhance both tumor treatment and prevention [179].

TLR9 is an important membrane protein that primarily detects pathogens by recognizing unmethylated CpG DNA sequences. Upon recognition of CpG DNA, TLR9 activates signaling pathways that lead to the production of cytokines, thereby initiating an innate immune response [180]. Chen et al. designed a self-adjuvanting system based on spherical nucleic acids (SNAs) composed of phosphodiester oligonucleotides and vitamin E. This system enhances TLR9 activation and serves as an effective anti-cancer vaccine. In tumor allograft models expressing OVA, the vaccine significantly delays tumor growth and extends animal survival, whether administered prophylactically or therapeutically. Additionally, it notably reduces lung metastases in the B16F10-OVA model [181]. Wang et al. developed an intelligent TME-responsive nanorobot composed of a matrix metalloproteinase 2 (MMP2)-cleavable GPLGVRGS motif and an arginine-rich GRRRDRGRS sequence. This nanorobot, which can effectively deliver CpG payloads to TLR9-positive tumors, acts as an adjuvant commonly used in cancer vaccination. It induces autophagy-mediated cell death for immunotherapy and can reprogram the tumor immunosuppressive microenvironment, thereby inhibiting tumor growth and recurrence [182].

### Cancer Nanovaccines Targeting T Cells

The first step in cellular immune response involves T cells recognizing and binding to antigen peptide-MHC complexes on APCs via their TCRs. Upon antigen recognition, T cells are activated and begin to proliferate and differentiate into various types of effector T cells, including helper T cells (Th cells), cytotoxic T cells (Tc cells), and regulatory T cells (Treg cells). Th cells are primarily categorized into Th1 and Th2 subsets. Th1 cells activate macrophages through the secretion of cytokines such as IFN-γ, enhancing their pathogen-killing ability, whereas Th2 cells promote B cell proliferation and antibody production by secreting cytokines such as IL-4 and IL-5. Tc cells directly kill virus-infected or tumor cells. Some activated T cells differentiate into memory T cells, which can respond more rapidly and effectively upon re-exposure to the same antigen, providing quicker immune protection. Treg cells maintain immune system balance by suppressing the activity of other immune cells, thus preventing the occurrence of autoimmune diseases [183, 184].

**Fig. 6:**
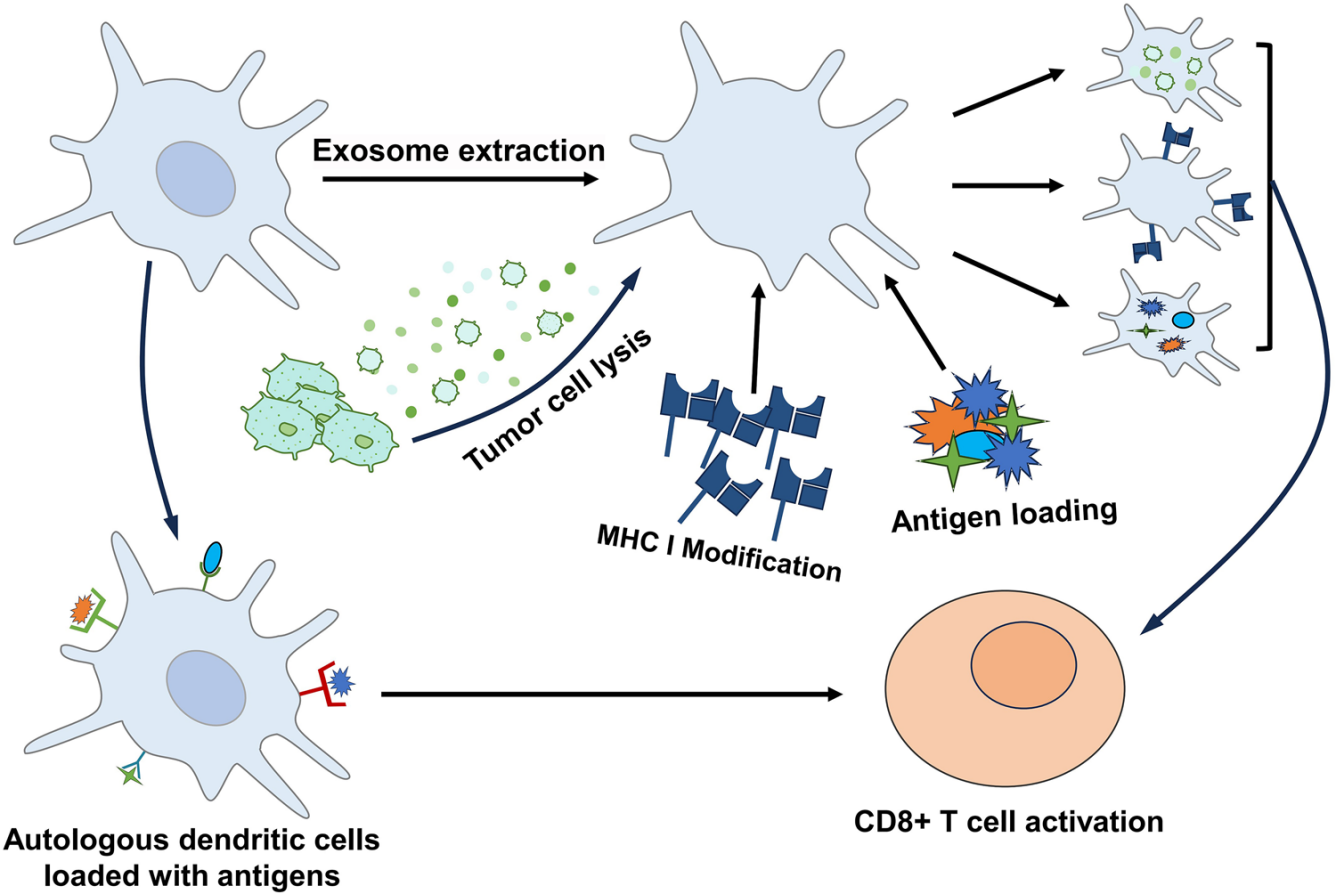
Cancer nanovaccines directly targeting T cells consist of two types of DCs. One type utilizes autologous DCs loaded with antigens to directly elicit T cell responses, while the other induces T cell responses through receptor modification and antigen loading using biomimetic dendritic cell membranes or dendritic cell-derived vesicles

Recent studies have shown that utilizing a strategy of antigen encapsulation with biomimetic dendritic cell membranes can directly target T cells in vivo, bypassing the need for antigen-presenting cell activation within the body (Fig. 6). Compared to dendritic cell-based vaccines, T cell-targeted vaccines can enhance the efficiency of immune responses, exhibit potent tumor-specific immune reactions, demonstrate lymph node homing capabilities, and induce long-term immune protection through memory T cells. These attributes make T cell-targeted vaccines promising for personalized cancer immunotherapy [185]. For example, some studies have utilized biomimetic dendritic cell nanovesicles (DCNVs) to encapsulate various antigens, such as mutant neoepitopes M27 and M30 on the surface of B16F10 and tyrosinase-related protein 2 (TRP2), and directly deliver them to T cells, thereby effectively combating melanoma [186]. A different research project attached IL-15Rα and complexes of tumor-associated antigen/major histocompatibility complex (TAA/MHC) to the mem-brane vesicles of genetically modified DCs, directing IL-15 specifically to CTLs that recognize the antigen and prolonging the circulation time of cytokines, thereby promoting the therapy of breast cancer, colorectal cancer, and melanoma model mice [187]. Additionally, researchers have developed a biomimetic dendritic cell nanovesicle (CSD) vaccine utilizing encapsulated Cu_2-x_Se nanoparticles (CSNPs) to mimic mature DCs. This vaccine is rich in highly expressed specific TAAs and possesses potent homing ability to lymph nodes. Moreover, the vaccination can enhance the release of TAAs from DC lysosomes via the MHC I pathway and simultaneously release small amounts of copper ions, which in turn accelerates the proliferation of T cells. These vaccines show enormous potential in treating highly infiltrative glioblastoma and highly metastatic melanoma [18]

### Nanovaccines Directly Targeting Cancer Cells

Due to genetic mutations, epigenetic changes, aberrant signaling pathways, and dysregulated cell cycle, cancer cells exhibit significant differences in morphology and protein expression compared to normal cells [188–190]. The irregularities result in changes in the presentation of numerous proteins on the exterior of cancerous cells [191–193]. Targeting and regulating these overexpressed receptors can facilitate the direct delivery of cytotoxic drugs, antigens or adjuvants to cancer cells. After drug-induced cell death, dying tumor cells express calreticulin on their surface and release endogenous damage-associated molecular patterns (DAMPs), such as high mobility group box 1 (HMGB1) and adenosine triphosphate. These molecules mediate the activation of DCs, leading to the expansion of tumor-specific T cells that eliminate residual tumor cells. When used in combination with adjuvants, tumor antigens, immune checkpoint inhibitors, and other agents, this type of vaccine can further activate the immune system, leading to effective short-term eradication of cancer and the maintenance of long-term immune memory against tumor recurrence and metastasis [194–197]. Therefore, we hereby introduce the concept of “Nanovaccines directly targeting cancer cells”, and this concept doesn’t focus on traditional preventive vaccines, but primarily on therapeutic vaccines.

#### SR-B1 on *Cancer* Cells

**Fig. 7:**
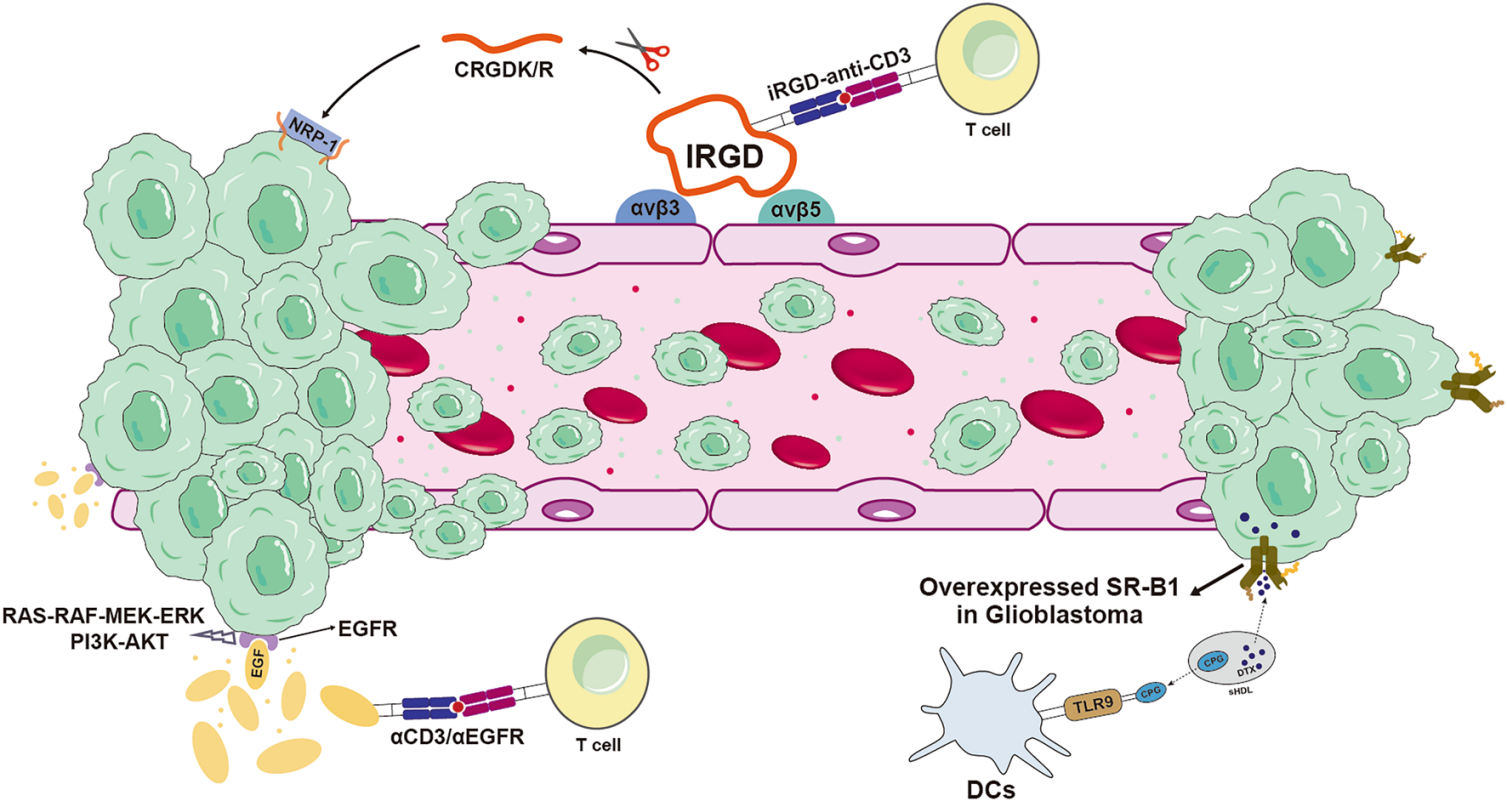
iRGD can bind to the highly expressed α_v_β_3_ and α_v_β_5_ integrins on the vasculature within tumor tissue, enzymatically cleaving to generate CRGDK/R which binds to NRP-1, facilitating drug delivery across the cancer cell membrane. Additionally, some cancer cell membranes exhibit overexpression of protein receptors such as EGFR and SR-B1, serving as targeting sites for cancer cells

SR-B1 expression levels vary across different cellular tissues and have been demonstrated to be abnormally overexpressed in certain tumor cells, playing crucial roles in the recognition, binding, and uptake of both endogenous and exogenous ligands (Fig. 7) [151]. Targeting SR-B1 for delivering cytotoxic drugs can effectively eradicate tumors, and especially when combining with the co-delivery of antigens and adjuvants, this strategy is also armed with vaccine-like properties. For instance, SR-B1 has been studied as a therapeutic target for glioblastoma [198]. In this context, Kadilyala et al. utilized sHDL nanodiscs as supports to load the TLR9 agonist CpG and the chemotherapeutic drug, docetaxel (DTX) and obtained a chemo-immunotherapy delivery system known as DTX-sHDL-CpG, and such system was specifically designed to target glioblastoma multiforme (GBM). When DTX killed cancer cells, CpG activated DCs within TME to process tumor released antigens and lead to the expansion of tumor-specific T cells. Ultimately, these activated T cells migrated to GBM, eliminating residual tumor cells, thereby achieving effective anti-glioma immunity and maintaining long-term immune memory against GBM metastasis [199].

#### α_v_β_3_ and α_v_β_5_ Integrins on Tumor Vasculature

The tumor vasculature surface contains the α_v_β_3_ and α_v_β_5_ integrins. They play crucial roles in tumor angiogenesis and maintenance, as well as tumor migration and invasion. Due to their abundant expression on the tumor vasculature surface, α_v_β_3_ and α_v_β_5_ integrins have become important targets for drug targeting therapy [200, 201]. The tumor-penetrating peptide iRGD is a cyclic peptide composed of nine amino acids, which can bind with above two integrins, and then produce CRGDK/R through enzymatic cleavage to interact with neuropilin-1, thereby facilitating targeted drug penetration into tumor tissues [202, 203]. A study developed a bifunctional agent, iRGD-anti-CD3, where anti-CD3 mediated iRGD to anchor to the surface of T cells, enhancing T cell infiltration into tumor tissues while coincidently inducing T cell activation and cytotoxicity against target cancer cells. This strategy demonstrated potent antitumor effects and generated long-term immune memory when used in combination with a nano-carrier delivering anticancer drugs and anti-PD-1 [204]. Another study fused a superantigen mutant, ST-4, with the tumor-homing peptide iRGD to construct the recombinant protein ST-4-iRGD. This approach effectively activated T cells within melanoma tissues, achieving strong tumor targeting and cytotoxicity. When combined with drugs in the future, this method could potentially produce even more powerful antitumor effects and immune responses [205].

#### Epidermal Growth Factor Receptor (EGFR)

EGFR, as a member of the tyrosine kinase receptor family, it is located within the cell membrane. When it binds with epidermal growth factor (EGF) and related molecules, it triggers downstream signaling pathway activations like RAS-RAF-MEK-ERK and PI3K-AKT, which in turn control cell proliferation, migration, and survival [206–208]. Abnormal EGFR expression and EGFR-mediated signal activation have been found in many human malignancies, wherein hyperactivated EGFR has been identified to be associated with the onset and progression of various tumors [207]. Therefore, targeting the highly expressed EGFR on tumor surfaces is also a strategy for developing cancer nanovaccines.

Cheng et al. designed a nanotherapeutic formulation named SMART-Exos, utilizing anti-EGFR and anti-CD3 antibodies produced by HEK 293 cells. The αCD3/αEGFR SMART-Exos target T cells through αCD3 and subsequently direct these T cells to EGFR-overexpressing breast tumor tissues via αEGFR, triggering effective antitumor immunity and generating immune memory to manage tumor progression [209]. Objective to those tumors with low EGFR expression, physical treatment such as ionizing radiation has been documented to not only directly kill tumor cells, but also upregulate EGFR expression, which, thereby, enabled EGFR blockade therapy and EGFR-targeted nanovaccines therapy against EGFR-negative cancer [210]. As a paradigm, Alghamri et al. developed synthetic protein nanoparticles (SPNPs) loaded with the CXCR4 antagonist AMD3100 to systemically target the CXCL12/CXCR4 axis in GBM. By blocking CXCL12/CXCR4 signaling, they found that GBM proliferation was inhibited, while immunogenic cell death (ICD) was induced, sensitizing the tumor to radiotherapy and triggering an anti-GBM immune response. Notably, their study showed that when AMD3100-SPNPs were combined with ionizing radiation therapy, T cells produced more effector molecules (GzmB and IFN-γ), and more than half of the experimental mice remained in tumor-free state even though they were rechallenged with GBM [211]. This result indicates that the activated adaptive immune responses prevented secondary tumor inoculation, harvesting the prolonged survival rate and enhanced immune memory which is crucial for eradicating resistant and recurrent GBM.

### Nanovaccines in Combination with Immune Checkpoint Blockade (ICB)

Immune checkpoints, which are immune inhibitory molecules found on immune cells, play a vital role in controlling immune activation to prevent overactive immune reactions [212, 213]. However, tumors exploit these immune inhibitory molecules to evade immune attacks. A transmembrane protein known as programmed cell death protein 1 (PD-1) is present on the surface of T cells. When PD-1 binds to its ligands PD-L1 or PD-L2 on the surface of tumor cells, it transmits inhibitory signals that reduce T cell proliferation and function. This interaction leads to T cell exhaustion, impairing the cells’ ability to effectively kill tumor cells and allowing tumor cells to evade immune surveillance [214, 215]. A similar mechanism is also present in the cytotoxic T lymphocyte antigen 4 (CTLA-4) pathway. CTLA-4 is an inhibitory receptor on the surface of T cells, expressed early during T cell activation, and competes with CD28 for binding to B7 molecules (CD80/CD86), thereby suppressing T cell activation. Tumor cells can exploit this pathway by expressing B7 molecules to inhibit T cell activity through CTLA-4 [216]. ICIs are a class of drugs that restore T cell activity by blocking the interactions between inhibitory receptors on T cells and their ligands. By inhibiting the binding of PD-1 to its ligands and the binding of CTLA-4 to B7 molecules, ICIs restore T cell anti-tumor activity, relieve suppression of T cells, and enhance T cell functionality [217].

Lately, the combination of cancer nanovaccines and ICB therapy has demonstrated notable effectiveness in diverse cancer therapies. An example of this is the creation of a biodegradable versatile vaccine utilizing poly(lactic acid) (PLA) nanoparticles, which was designed to simultaneously transport intracellular antigens related to breast cancer, TLR ligands, and small interfering RNA (siRNA) aimed at transforming growth factor-β1 (TGF-β1), ultimately increasing responsiveness to OX40 immune checkpoint agonist and encouraging T cell anti-tumor response [218]. Another study’s nanovaccine (R837@HM), mesoporous silica nanoparticles (MSN) as a delivery vehicle, combined with DC-cancer cell hybrid membrane and R837 immune adjuvant to form a novel hybrid membrane nanovaccine. The vaccine, in conjunction with αPD-1, greatly boosted the combined effect of halting tumor growth, eradicating existing tumors, and resisting tumor rechallenges by altering the immune-suppressive environment, encouraging anti-tumor immune reactions, and triggering immune memory effects [219]. In addition, a group of researchers created nanovaccines made of tannic acid (TA) and recently designed protein antigens, with the use of IFN-α or CpG as adjuvants. The vaccination boosted the body's defenses against tumors linked to the Ep-stein-Barr virus, ensuring more effectively treatment consequences when used along-side anti-PD-L1 therapy. In experiments with combination therapy using low-dose an-ti-PD-L1, approximately 70% of tumors completely regressed, whereas the tumor regression rates with anti-PD-L1 or NA1C monotherapy were only about 10% and 30%, respectively [220]. The article discusses different nanovaccines paired with ICB therapy [19, 20, 221] suggesting that combining the two treatments could improve the effectiveness of cancer nanovaccines.

### Hybrid Cell Nanovaccines

The advancement of cancer nanovaccines is mainly dependent on antigen-presenting cells, like DCs, to stimulate T-cell immune reactions for the eradication of tumors [23, 222, 223]. Nevertheless, B lymphocytes, crucial for antibody-mediated immunity, could also contribute to eliminating tumors. To effectively treat and prevent infectious diseases, it is often essential to stimulate both B cell and T cell immunity simultaneously in order to produce long-lasting and powerful immune responses [224–228]. The tactic could also be beneficial in the treatment of cancer.

**Fig. 8:**
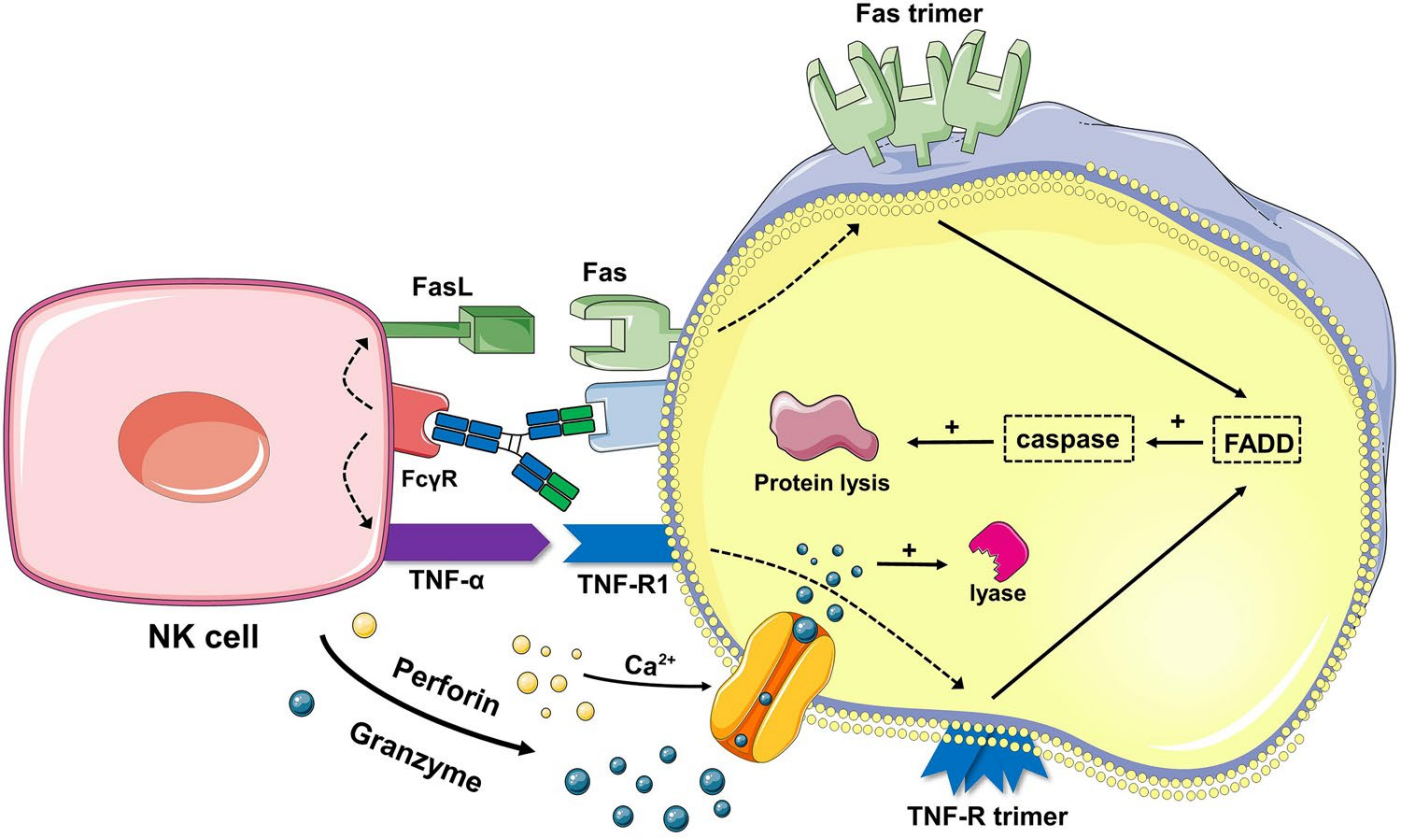
Diagram of the ADCC mechanism. When the Fab segment of the antibody binds to receptors on the target cell, the Fc segment receptor on NK cells binds to the Fc segment of the antibody, triggering the NK cells to release TNF and engage FasL with Fas, inducing the formation of TNF-R and Fas trimers on the target cell. This activation leads to the activation of caspase proteins within the target cell, cleaving intracellular proteins. Simultaneously, NK cells release perforin and granzyme, which activate caspases. Both processes work concurrently to kill the target cell

Some antibodies can directly kill tumor cells by targeting receptors or delivering drugs and cytotoxic agents. Others indirectly kill tumor cells by mediating the actions of other immune cells or complement [229]. Notably, antibody-dependent cellular cytotoxicity (ADCC) involves natural killer (NK) cells. Specific antibodies bind to antigens on the surface of tumor cells, and the Fc receptors (FcγRIIIa) on NK cells bind to the Fc region of these antibodies. This activates NK cells to release cytotoxic substances such as perforin and granzymes, which disrupt the structure of cancer cells, leading to direct cell killing (Fig. 8) [230]. Monocytes, macrophages, and other phagocytic cells participate in antibody-dependent cellular phagocytosis (ADCP). In this process, antibodies bind to tumor cells, and the Fc receptors (such as FcγRIIa, FcγRI, and FcγRIIIa) on effector cells bind to the Fc region of the antibodies. This activates the effector cells to internalize the target cells through phagocytosis, enclosing them in phagosomes. These phagosomes then fuse with lysosomes, releasing enzymes and acidic substances that degrade the components of the target cells, resulting in the death of cancer cells [231]. Complement-dependent cytotoxicity (CDC) involves the complement system. When antibodies bind to tumor cells, they activate the complement system, leading to the formation of membrane attack complexes that cause tumor cell lysis. Additionally, some antibodies enhance the immune system’s attack on tumor cells by blocking immune checkpoints. These various strategies can all serve as the basis for designing cancer nanovaccines [232].

**Fig. 9:**
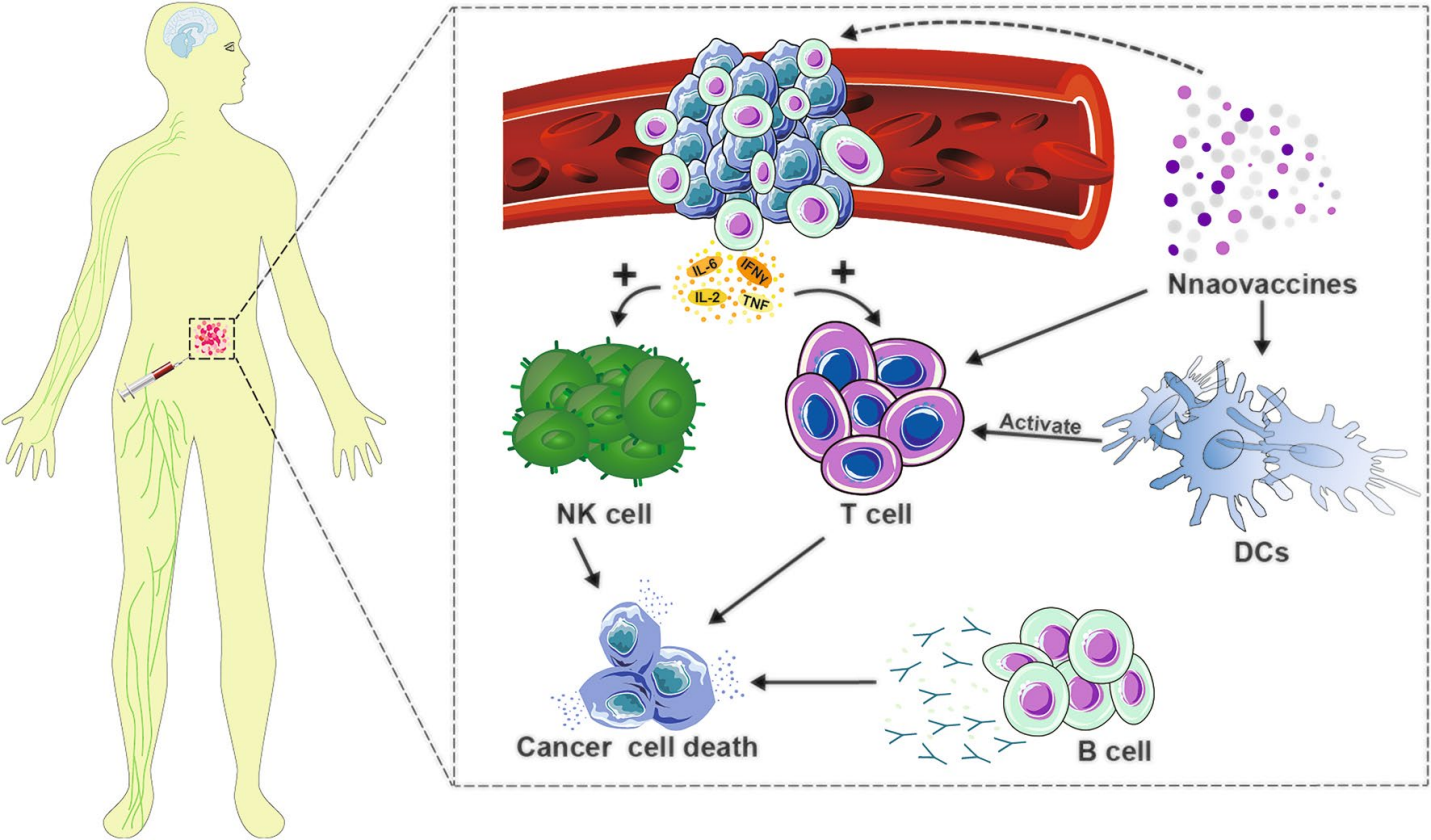
Within the vasculature of tumor tissue, B cells can generate proinflammatory factors to activate immune effector cells. Hybrid nanovaccines can facilitate this process in synergy with antibody-mediated cytotoxicity against cancer cells

New research has shown that activated B cells within tumors can enhance the display of antigens and release inflammatory cytokines (such as TNF, IL-2, IL-6, and IFN-γ), which in turn triggers the activation and recruitment of immune effector cells like CD4 + and CD8 + T cells. The activation and expansion of these cells further promote the immune response of specific T cells, thereby enhancing the immune effect against tumors [233–236]. Numerous research groups are focused on creating a cancer nanovaccine that can stimulate B cells, T cells and NK cells at the same time for longer-lasting and more powerful anti-tumor results, known as a hybrid cell nanovaccine (Fig. 9).

Gul et al. created an innovative DNA vaccine containing antigenic epitopes (Me-Her2) for HER2 proteins, along with an antibody fragment that targets DCs uptake receptor DEC205 (ScFvDEC). By constructing this protein, the vaccine is designed to contain numerous T cell epitopes, B cell epitopes, and ScFvDEC, which helps boost its ability to trigger an immune response [21]. Wang et al. employed folate (FA)-encapsulated liposomes for the administration of ovalbumin (OVA) and TLR4 stimulant MPA, creating a nanovaccine FA-sLip/OVA/MPLA. This vaccine was successfully delivered to splenic marginal zone B cells, triggering immune responses from both humoral and CTLs, resulting in a notable slowdown of tumor growth in the E.G7-OVA tumor mouse model. It was also found that its combination with anti-PD-1 therapy improved anti-tumor efficacy [19]. Li et al. developed an antigen cluster nanovaccine ACNVax by linking iron nanoparticle cores with appropriate gold nanoparticles, loaded with HER2B/CD4 T cell epitope clusters. ACNVax successfully induced extended tumor regression through enhancing migration to lymph nodes and cross-priming of B/CD4 T cells. Specifically, when paired with an anti-PD-1 antibody, ACNVax resulted in more than 200 days of extended tumor regression in the HER2-positive breast cancer mouse model, boasting an 80% rate of complete remission [222], while concurrently inducing cell-mediated immunity.

Furthermore, a new research project employed 8 M urea to dissolve components that are not soluble in water produced from lysed cancer cells or tumor tissues. These components were then loaded onto nanovaccines, along with water-soluble components, greatly improving the nanovaccines’ capacity to stimulate antigen-specific T cells and boosting the presence of B cells to encourage the development of tertiary lymphoid structures at tumor locations. This vaccine achieved prevention and cure rates of 100% and 70% for lung cancer and melanoma, respectively, and effectively treated melanoma and triple-negative breast cancer [22]. Research suggests that utilizing hybrid cell nanovaccines to treat tumors is possible, and they demonstrate enhanced tumor-killing capabilities when paired with specific compounds that enhance T-cell function (like anti-PD-1 antibodies).

## Application of *Cancer* Nanovacciens

Currently, for various highly prevalent human cancers such as AML [106], melanoma [18, 22, 84, 95, 143, 150, 171, 186], breast cancer [22, 95, 101, 143, 204, 221], liver cancer [198], nasopharyngeal carcinoma [195, 210], lung cancer [22], colorectal cancer [95, 172, 205], glioblastoma [18, 171, 196], and human papilloma-virus-related cancers [98], animal experiments have demonstrated significant efficacy of cancer nanovaccines in antitumor therapy. However, due to the complexity of nanomaterial design and fabrication processes, along with prolonged cycles of validation and modification, most cancer nanovaccines face challenges in cost control and quality assurance, making their path to clinical application still difficult. Currently, cancer nanovaccines that have entered clinical trials or applications mainly utilize designed nanomaterials to deliver drugs such as paclitaxel and glycyrrhizic acid directly inducing tumor cell death, or encapsulate immune checkpoint monoclonal antibodies to relieve T cell suppression and thereby induce tumor cell death (Table 1).

**Table 1:** A summary on current progress of cancer nanovaccines in clinical trials

Cancer type	Nanocarriers	Combined drugs	Phase	TAA/TSA	Current status	Trial number
Non-small lung cancer (NSLC)	Lipid nanoparticles	Pembrolizumab	I/II	DNA plasmid/TUSC2 tumor suppressor gene	Recruiting	NCT05062980
Pediatric solid tumors	Albumin-bound nanoparticles	Rapamycin/ temozolomide/ irinotecan hydrochloride	I	–	Not recruiting	NCT02975882
Advanced solid tumors	Carbon nanoparticlen	Iron [CNSI-Fe (II)]	I	–	Recruiting	NCT06048367
TSCC	Polymeric nanoparticles	Quercetin	II	–	Not recruiting	NCT05456022
Metastatic breast cancer	Albumin-bound nanoparticles	Paclitaxel	II	–	Not recruiting	NCT00609791
Glioblastoma	Polysiloxane Gd-Chelates-based nanoparticles	Temozolomide	I/II	–	Recruiting	NCT04881032
Pancreatic cancer	Protein bound-nanoparticles	Ascorbic Acid/ Cisplatin/Gemcitabine/ Paclitaxel	I/II	–	Completed	NCT03410030
HGG	Dendritic cells	Nivo/Ipi double checkpoint blockade/Nivolumab	I/II	Tumor-lysate	Recruiting	NCT03879512
Uveal melanoma	Dendritic cells	–	III	Tumor RNA	Not recruiting	NCT01983748
Acute myeloid leukemia	Dendritic cells	–	Not Applicable	WT1/hTERT/Survivin	Recruiting	NCT05000801
Malignant tumors	Dendritic cells	ICIs	I	TP53 Mutant Peptide	Recruiting	NCT05631886

Paclitaxel and eribulin have been utilized in clinical settings, with ongoing clinical trials for breast cancer (NCT00609791) and head and neck cancer (NCT01847326) involving paclitaxel. In addition to conventional treatment strategies, some novel studies utilize iron oxide particles to prepare vaccines and have entered clinical trials. For example, Trujillo-Alonso et al. administered ferumoxytol as a vaccination to enhance intracellular iron levels, control internal iron processing routes, generate surplus reactive oxygen species (ROS), trigger oxidative stress, and induce toxicity in cancerous cells [237]. Zanganeh et al. discovered that ferumoxytol has the ability to hinder tumor progression by triggering M1 macrophage-induced inflammatory reactions in tumor areas and blocking the formation of metastases in the liver [47]. Additional studies have indicated that a combination of carbon and iron particles can decrease the harmful effects of injecting pure iron oxide particles [238]. This approach, using carbon nanoparticles containing iron [(CNSI-Fe (II)], is now being tested in phase I clinical trials for treating advanced solid tumors (NCT06048367). A new clinical trial has been updated to include the Epstein-Barr virus (EBV) gp350-ferritin nanoparticle vaccine (NCT04645147) since EBV is linked to diseases like Hodgkin’s lymphoma, non-Hodgkin’s lymphoma, gastric adenocarcinoma, nasopharyngeal carcinoma, aggressive NK cell leukemia, and peripheral T cell lymphoma [239]. The study aims to balance safety and antigenicity to facilitate safe use in the future prevention of EBV-related cancers. In addition, reviews and the latest clinical trial data suggest that the combination of cancer nanovaccines mentioned above with ICB therapy may enhance the efficacy of tumor treatment [240]. The efficacy of mRNA nanoparticle vaccines in combination with PD-1 blockade therapy is being evaluated in a recent phase I clinical trial (NCT03739931) to determine the optimal drug dosage and administration method, with potential implications for future vaccine treatment strategies. A recent clinical trial (NCT02716012) is exploring the use of MTL-CEBPA to boost C/EBP-α levels in order to eradicate tumors through immune checkpoints. Approximately 70% of the 34 advanced liver cancer patients who completed treatment showed effective therapeutic effects [241]. In addition to this, cancer nanovaccines directly targeting T cells have also emerged in clinical trials. These trials employ a biomimetic nanomaterial carrier strategy to induce an in vivo T cell response, i.e., utilizing DCs to encapsulate antigenic components. For example, trials have given patients with uveal melanoma DCs that contain their own tumor RNA (NCT01983748). A different experiment includes the attachment of peptides derived from tumor blood vessel antigen (TBVA) onto the patient's own DCs, which are then given to individuals with localized clear cell renal cell carcinoma. This treatment is paired with cabozantinib therapy in order to achieve the elimination of cancer effects (NCT05127824).

The potential for personalized nanovaccines tailored to the unique tumor characteristics of individual patients remains a significant area for exploration. Therapeutic approaches targeting specific monotypic tumors can substantially enhance efficacy and reduce side effects, making it a key area for future research. Moderna is developing a personalized cancer vaccine named mRNA-4157/V940 (NCT03897881). This novel antigen-based mRNA vaccine is designed and produced based on the mutation profile of a patient’s tumor, targeting patient-specific mutations and encoding up to 34 neoantigens. Moderna is currently advancing a combination therapy of mRNA-4157/V940 with Merck’s anti-PD-1 therapy Keytruda for adjuvant treatment in high-risk melanoma patients. This combination therapy has been shown to reduce the risk of recurrence or death by 44% compared to Keytruda alone, offering new hope for personalized neoantigen cancer treatment. Similarly, research based on mRNA-4157 will undergo a Phase I clinical trial targeting solid tumors and exploring the efficacy of combination monoclonal antibody therapy (NCT03313778). The company is also conducting two other clinical trials for mRNA vaccines: one based on mRNA-4359 for advanced solid tumors is currently recruiting participants (NCT05533697), while another trial based on mRNA-2416 was terminated due to not meeting the expected efficacy (NCT03323398). BioNTech and Genentech have jointly developed an mRNA vaccine, Autogene cevumeran, for the treatment of pancreatic cancer. Results from a Phase I clinical trial indicate that in some patients, immune cells activated by the mRNA vaccine remain in the body for up to three years post-treatment. The immune response induced by the vaccine is associated with a reduced risk of cancer recurrence, suggesting that the vaccine-activated T cells may recognize and attack pancreatic cancer as foreign cells. The Phase II clinical trial of Autogene cevumeran has been launched under the sponsorship of BioNTech and Genentech, with a global recruitment target of 260 patients (NCT05968326). Immatics Biotechnologies GmbH is conducting a clinical project named the “Glioma Actively Personalized Vaccine Consortium (GAPVAC).” This project, in its Phase I trial, tested a personalized vaccine comprising non-mutated TAAs and neoantigens. The results showed that the non-mutated APVAC1 antigens activated sustained central memory CD8 + T cell responses, while APVAC2 predominantly elicited CD4 + Th1 responses targeting predicted novel epitopes (NCT02149225) [242].

## Challenges and Outlook

Admittedly, we have observed significant anti-tumor effects of cancer nanovaccines in animal experiments, and there have been some clinical application trials. Despite the exciting achievements of cancer nanovaccines, there remain significant challenges and many unresolved issues that need to be addressed in translation from animal experiments to clinical trials. Therefore, there is still a long way to go before they can be fully implemented in clinical practice.

### Potential Toxicity and Long-Term Safety Concerns

For nanoparticles, their composition, assembly methods, particle surface, ligands, rigidity, and charge can all affect their performance. The complex composite structures make it difficult to analyze the potential toxicity of nanoparticles. Before clinical application, it is often necessary to assess the interactions between nanoparticles and the human body to determine their potential toxicity. However, a suitable biological model to evaluate these interactions has not yet been developed [243].

Some studies have revealed the harmful effects of cancer nanovaccines currently used in animal experiments on the body. Metal nanoparticles may induce metal deposition and oxidative stress, while viral vectors may cause viral infections because of insufficient inactivation. ROS have been identified as a major cause of cytotoxicity. Metal materials such as gold and iron oxide, and inorganic materials like silica, have been shown to generate ROS within the body, leading to low biocompatibility. To better address the issue of ROS generation, merely quantifying the ROS levels induced by materials is insufficient. Understanding the mechanisms behind ROS production and the associated oxidative stress is essential to achieving broader goals for the safety of nanoparticles [244]. For example, Lehman et al. found that the increased porosity of MSN and amine functionalization of nanoporous silica nanoparticles reduced ROS production at the solid-liquid interface. This indicates that ROS generation can be controlled by altering surface properties and porosity [245]. Li et al. simulated the ROS generation kinetics of various metal nanoparticles and elucidated the mechanisms of ROS production by interpreting their electronic structures. They discovered that ROS generation by metal oxides is pH-dependent. Adjusting conditions such as pH and bandgap may help design an optimal injection environment for cancer nanovaccines [246].

In addition to ROS production, other cancer nanovaccines with good biocompatibility may also have potential toxicity, which can arise from the carrier and antigen materials. For instance, several studies have found that injecting mRNA vaccines into mice can cause mild toxicity to the liver and spleen, leading to a reduction in lymphocytes. Research has shown that mRNA vaccines delivered by lipid carriers can increase pro-inflammatory cytokines such as IL-6 in mice, and LNP-mRNA formulations can also activate the complement system. Although rare, complement activation may lead to allergic reactions, which, while uncommon in vaccination, can result in serious consequences such as myocarditis and laryngeal edema. Although lipids are frequently used as biocompatible nanovaccine carriers, some reports indicate potential toxicity. For example, simply injecting lipid carriers without the mRNA can also result in the secretion of pro-inflammatory factors, suggesting that lipid carriers themselves may be a source of mRNA vaccine toxicity. Ionizable lipids may bind to PRRs and initiate innate immune responses, producing harmful unsaturated fatty acids and causing intracellular lipid peroxidation. Additionally, lipids coated with PEG have potential immunotoxicity due to their uncertain immunogenicity, which requires further scrutiny [247]. This indicates that mRNA vaccines may potentially induce systemic inflammatory responses, such as cytokine storms and allergic reactions.

Taken all above together, enhancing biocompatibility in the human body and determining the appropriate vaccine dosage are key challenges that need to be overcome in the future construction of nanocarriers. Allergen test is also suggested before use. In particular, many anti-tumor effects achieved in animal experiments have not yielded ideal results in human experiments, highlighting the importance of constructing humanized animal models. Different expression patterns of C-type lectin receptors, TLRs, and scavenger receptors targeted by vaccines in mice suggest that murine vaccines may not be effective in humans. We believe that transplanting human tumor cell lines cultured in vitro into immunodeficient animals or inserting human genes into animals to replace their endogenous genes through gene-editing techniques, are viable methods for modeling human tumor diseases. For personalized diseases, patient tissue xenotransplantation can be employed to construct animal models homologous to patients, enabling more precise disease treatment.

### Reliable, Economical and Stable Mass-Production: from Laboratory to Clinic

The successful anti-tumor effects of one cancer nanovaccine in experimental animals do not mean it can be easily translated to humankind. In fact, translation from laboratory to clinic needs to overcome the challenges in stable mass-production technology and preclinical validation. Currently, high labor costs and material expenses pose significant obstacles, and standardizing product quality remains difficult. When the production process of nanoparticles involves multiple complex steps or technologies, achieving high reproducibility and transparency becomes more challenging. Additionally, due to the inherent differences between experimental animals and humans, the translation from laboratory to clinical settings always involves optimization of formulation parameters or even changes in manufacturing methods. Such optimization and changes often require substantial costs and innovative approaches, rendering many promising studies halted before clinical translation. To improve clinical translation rates, researchers should engage in prospective planning during animal experiments and develop strategies for nanoparticle design and production in advance.

Advanced technologies for large-scale production of nanoparticles have been developed. PRINT (Particle Replication in Non-Wetting Templates) technology is suitable for the reproducible manufacturing of nanoparticles, but scaling it up to kilogram quantities still needs to be demonstrated [248]. A recently developed coaxial turbulent jet mixer technology offers advantages such as uniformity, reproducibility, and adjustability, which are typically only achievable with microfluidic or micro-scale mixing techniques. This technology is used for the large-scale production of polymer nanoparticles, with a potential throughput of 3 kg per day per channel [249]. Although batch synthesis remains the cornerstone of nanoparticle production, robust and versatile methods such as PRINT and turbulent jet mixer technologies can prepare nanoparticles at industrial-scale throughput, potentially accelerating clinical translation. Determining optimal physicochemical parameters is crucial for the successful development of therapeutic nanoparticles. However, due to the difficulty in rapidly, accurately, and reproducibly synthesizing nanoparticles with varying properties, ensuring reproducibility and transparency severely limits systematic and large-scale screening of nanoparticles.

### Combined Therapy

In recent years, we have witnessed the tremendous impact of immunotherapies such as CAR-T therapy, ICB therapy and other nanobiotechnology-enhanced immune therapy in cancer management [250–252]. As a promising novel immuno-therapeutic strategy, cancer nanovaccines have demonstrated remarkable prophylactic and therapeutic effects against tumors in animal experiments. Additionally, recent clinical studies have demonstrated the effectiveness of cancer nanovaccines in treating cancer patients. It has been widely accepted that the ultimate efficacy of vaccines depends on their ability to elicit strong immune responses. Although nanovaccines themselves are more likely to induce immune responses than traditional vaccines due to their small size, the immunogenicity of vaccines still needs to be improved based on the therapeutic effects achieved by current cancer nanovaccines. At present, it is unrealistic to expect a 100% tumor cure rate solely from cancer nanovaccines. Inspiringly, combined therapy strategies are highlighted and arouse increasing attentions. The combination of cancer nanovaccines with chemotherapy, ICB, other physical therapy and external administration of tumor-killing agents shows the potential to improve the effectiveness of vaccines [253–256]. Vaccine adjuvants can enhance vaccine immunogenicity, so developing new adjuvants or using novel methods to modify nanomaterials to confer adjuvant effects is a promising approach. Improving the precision of the vaccine's targeting will boost the presentation of antigens and stimulate T-cell immune reactions. Therefore, improving the targeting antibodies loaded onto the nanocarriers is also a method to address this issue.

In summary, cancer nanovaccines represent a highly promising cancer treatment modality. Despite not being ready for clinical use yet, advancements in materials science, biology, and immunology suggest that further studies on cancer nanovaccines will soon be conducted. Future cancer treatment is expected to rely on safer and more efficient nanovaccines as the primary therapy.
